# Mapping arealisation of the visual cortex of non-primate species: lessons for development and evolution

**DOI:** 10.3389/fncir.2014.00079

**Published:** 2014-07-04

**Authors:** Jihane Homman-Ludiye, James A. Bourne

**Affiliations:** Bourne Group, Australian Regenerative Medicine Institute, Monash UniversityClayton, VIC, Australia

**Keywords:** cortical patterning, guidance molecules, neocortex, cell markers

## Abstract

The integration of the visual stimulus takes place at the level of the neocortex, organized in anatomically distinct and functionally unique areas. Primates, including humans, are heavily dependent on vision, with approximately 50% of their neocortical surface dedicated to visual processing and possess many more visual areas than any other mammal, making them the model of choice to study visual cortical arealisation. However, in order to identify the mechanisms responsible for patterning the developing neocortex, specifying area identity as well as elucidate events that have enabled the evolution of the complex primate visual cortex, it is essential to gain access to the cortical maps of alternative species. To this end, species including the mouse have driven the identification of cellular markers, which possess an area-specific expression profile, the development of new tools to label connections and technological advance in imaging techniques enabling monitoring of cortical activity in a behaving animal. In this review we present non-primate species that have contributed to elucidating the evolution and development of the visual cortex. We describe the current understanding of the mechanisms supporting the establishment of areal borders during development, mainly gained in the mouse thanks to the availability of genetically modified lines but also the limitations of the mouse model and the need for alternate species.

## Introduction

The visual cortex, responsible for providing the visual sensory experience, is a feature common to all mammalian species however large or small. Located at the occipital pole of the brain the visual cortex receives, integrates and interprets the information relayed from the eye via subcortical nuclei.

Despite the seemingly homogenous appearance of the neocortical surface, the visual cortex is subdivided into cytologically and functionally unique modules, forming a mosaic of adjoining areas. Despite sharing the 6-layer organization of the neocortex, each visual area (cortice) exhibits a characteristic laminar cytoarchitecture with subtle differences in layer thickness and cell density, which enables cytological identification. The neuroanatomist Korbinian Brodmann took advantage of this attribute to map the neocortex of various species, utilizing Nissl substance (cresyl violet) staining to reveal the distinct areal borders within the cortical sheet. These maps (Brodmann, 1909) were the first evidence of the arealisation of the neocortex, and have been since refined with more sophisticated anatomical and functional mapping.

The visual message is complex and comprised of many features, including shape, color, speed or direction of a moving object, which are each processed in a dedicated visual area. The processing of the visual information is a stepwise process, with inputs first relayed from the thalamus to the primary visual area (V1) and from there sequentially despatched to “extrastriate” areas organized in a hierarchical fashion through reciprocal connections (Felleman and Van Essen, 1991). The highest order areas in the hierarchy receive a refined message and perform complex integrative and associative processing (Goldman-Rakic, 1988; Mountcastle, 1997). The basic principles of functional organization are relatively conserved across species, however the number of visual areas varies across species, depending on the priority placed upon vision as a source of sensory input. Additional areas allow for in-depth, refined processing providing a more elaborate representation of the visual scene.

Many groups using a variety of techniques and animal models, including rodents, primates and carnivores have been involved in defining their visual cortical maps, resulting in the evolution of diverse nomenclature systems. In his seminal study, Korbinian Brodmann numbered the cortices according to cytoarchitectural criteria (Brodmann, 1909), with V1 originally classified as area 17 and the second visual area (V2) classified as area 18. The emergence of electrophysiological techniques and functional mapping led to a method of nomenclature relating to the area’s role, such that the primary visual area gained its name V1. Other visual areas were named depending on their position relative to V1. This system rapidly proved limited as more interleaving areas were identified, and also because of the diverse brain morphologies it was difficult to correlate maps between species. Therefore, a new system was devised, based this time on the spatial position of the area on the cortical surface. Examples include area V5 in the primate, which also received the nomenclature–middle temporal area (MT). In addition, mouse V2 is often referred to as the lateromedial area (LM; Wang and Burkhalter, 2007; Wang et al., 2012). To date, there still does not exist a uniformed system, and the nomenclature varies at the authors’ discretion, giving opportunity to confusion. This is no clearer than when dispute occurs over different territories in the visual cortex, or when areas are subdivided.

The specific limits of each cortice have also been the cause of much dispute, often due to the approach used, as each method is based around a particular functional or anatomical property and it is difficult to reconcile maps obtained using distinct strategies. This is clearly illustrated in the visual cortical map of the mouse, a model in which somatosensory and olfactory systems dominate and the small brain size limits accurate electrophysiology mapping (Wagor et al., 1980). Two concurrent studies attempted to resolve this longstanding issue using separate methods. One mapped the cortical fields lateral to V1 and recipient of direct inputs from V1 connections, revealed by triple anterograde fluorescent tracing and electrophysiology (Wang and Burkhalter, 2007). The second was based on the expression of the cytoskeletal marker nonphosphorylated neurofilament (NNF), characterized by its area-specific profile in the visual cortex combined with neuronal activity markers (Van der Gucht et al., 2007). Both groups concluded on the existence of discrete extrastriate areas in the mouse neocortex, however the studies conflicted on the number and location of areas identified. The tracing study demarcated seven domains comprising a complete map of the entire visual field in the region lateral to V1, compared to two subdivisions revealed by early response genes and NNF immunoreactivity. This example highlights the difficulty to reconcile maps generated using distinct methodologies, although the multimodal nature of areas beyond V1 in the mouse adds a level of complexity.

Arealisation is not limited to demarcating the spatial plan of cortical areas; great efforts are put into understanding other aspects of arealisation, including the evolutionary events that have led to the emergence of new cortical areas in higher species during the expansion of the neocortical surface and why the addition of new areas is more advantageous than the enlargement of pre-existing ones. Major progress has been made in understanding the evolution of cortical areas by defining the visual maps of a large number of species on different branches of the phylogenetic tree and comparing the cortical organization, number of areas or relative position of areas fulfilling equivalent function. For example, the existence of two processing streams in the primate—the dorsal “where” and ventral “what” pathways (Mishkin and Ungerleider, 1982; Ungerleider and Mishkin, 1982; Kravitz et al., 2011), have also recently been purported to be a feature of the mouse visual cortex (Wang et al., 2012), suggesting that it is not exclusive to the primates and that it must have evolved much earlier in the evolution of the visual cortex.

A prerequisite to a comparative approach is the availability of a wide range of cortical maps including atypical species such as the monotremes (e.g., echidna) or the eusocial naked mole rat (Hassiotis et al., 2004; Matsunaga et al., 2011), which is sometimes difficult to achieve using electrophysiological mapping. Therefore, researchers have taken advantage of alternative properties of visual cortical areas to consistently define their borders including molecular and chemical markers, which allow the use of fixed brain tissue.

Molecular markers are extremely powerful at demarcating visual areas, including at early stages of development, essentially before eyes open or the visual system has begun to function. They have therefore prompted major progress in the field of embryonic arealisation, which addresses how the position and identity of individual areas are specified in the developing neocortex. At the onset of corticogenesis, cortical areas progressively acquire their positional identity under the influence of molecular regulators differentially distributed across the developing brain (for review see O’Leary et al., 2007). The potential of creating transgenic animals in which the expression of the cortical patterning factors is perturbed has contributed to the prominence of the mouse in the field.

In this review, we will detail the molecular markers routinely used to define visual cortical areas and the animal models in which this has been employed. We will then comment on the importance of non-primate maps in clarifying the evolutionary relationship between visual areas and cortical expansion. Finally, we will present the current understanding of the mechanisms and actors underlying the specification of areal borders, consisting mainly of studies performed in the mouse but also including recent data from other non-primate species.

## How are visual cortical areas defined?

**Figure 1:**
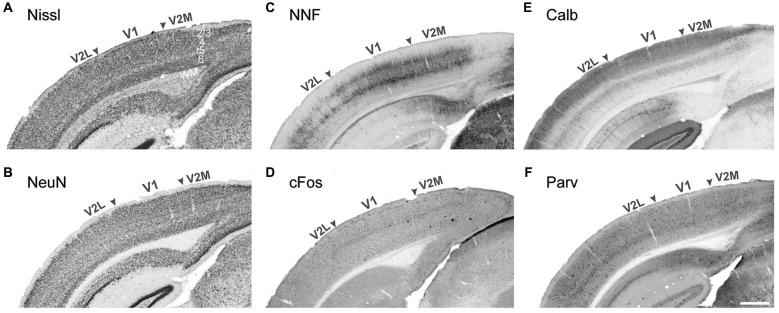
**Demarcation of the primary and secondary visual areas in the mouse adult neocortex using different markers**. Nissl cell staining **(A)** and the neuronal marker NeuN **(B)** are not sufficient to demarcate areal boundaries compared to pyramidal neuron marker nonphorsphorylated neurofilament (NNF, **C**) and the early response gene cFos **(D)**, strongly expressed in V1 compared to adjacent lateral and medial secondary visual areas (V2L and V2M respectively). The interneuronal markers Calbindin **(E)** and Parvalbumin **(F)** display strong laminar differences with a higher density of Calbindin+ cells in layers 2–4. Stronger Calbindin signal in V1 layer 4 is very efficient at demarcating the borders with adjacent secondary areas. *WM* white matter Scale bar in **(F)** 500 μm.

Visual areas can be characterized by many anatomical and functional features. The limiting factor has usually been the unavailability of tools to efficiently detect these specific features. Historically, the characterization of visual cortical organization has been achieved using simple cellular staining techniques, such as Nissl substance (cresyl violet) staining, which stains the rough endoplasmic reticulum, or labeling for the pan-neuronal transcription factor NeuN. The technique is extremely effective at demarcating cortical layers and therefore areas for which layer thickness and/or cell density vary markedly from their immediate neighbors (Figures 1A,B). This is specially the case in primates for early areas such as V1, as the cytoarchitecture of higher order areas is more homogenous in terms of their laminar pattern and cell number (Rockel et al., 1980). Therefore, for many years there has been an inability to accurately demarcate the extrastriate visual areas of most species. The application of new staining methods and the advance of antibodies technology has helped characterize more area-specific features enabling identification of discrete cortical nuclei. The techniques presented here are organized according to the specific feature they reveal.

### Connectivity

Individual areas establish a unique network of inputs and outputs with other cortical areas and subcortical domains. For V1, thalamic afferents form essentially glutamatergic synapses with layer 4 neurons (López-Bendito and Molnár, 2003). During development, thalamic neurons transiently uptake serotonin from the extracellular environment; the “borrowed” neuromodulator is then transported along axons to the neocortex, where it is then released in areas recipient of thalamocortical projections (Lebrand et al., 1996). Therefore, simple immunolabeling for the neurotransmitter is capable of accurately demarcating V1 in the mouse (Chou et al., 2013; Vue et al., 2013).

It is also possible to directly label the tracts using the physical properties of dyes that are transported along the axon from the cell body to the synapse (anterograde) or from the synapse to the cell body (retrograde). These tracers, largely fluorescent, can be used to map the connections emerging from an area of interest or the regions projecting onto the region of interest. This approach, recently utilized in the mouse (Wang et al., 2011) and the rat (Watakabe et al., 2012), can be combined with 3D modeling to provide details on the functional relationship between areas. Additionally, these paradigms can also be applied in developmental studies to determine when areas become wired together and therefore the relative hierarchy of individual areas (e.g., the establishment of thalamocortical connections in the mouse) (Little et al., 2009; Deck et al., 2013). Laramée et al. (2013b) used a combination of red anterograde and green retrograde fluorescent tracers in mice to investigate the consequences of visual deprivation (congenital anophthalmia and perinatal enucleation) on the topography of projections from V1 to extrastriate areas and callosal connections, revealing an important disorganization and reinforcing the importance of retinal input in the establishment of corticocortical circuits. The authors also investigated in the same mice the effect of early loss of sensory-driven activity on the afferent cortical and subcortical projections to V1 using retrograde tracer injection. They traced direct projections from the somatosensory and auditory cortices onto V1 in all three animal groups, demonstrating that multimodality is not a consequence of congenital/perinatal blindness (Charbonneau et al., 2012).

Finally, projections can also be traced by viral mediated expression of reporter proteins. For example, enhanced green fluorescent protein (EGFP) under the control of a neuron specific promoter, such as that for synapsin. The viral particles reach the cell by retrograde transport and express the reporter protein which then distributes into the dendrites and collateral (Tomioka, 2006). This robust Golgi-like stain allows the reconstruction of the dendritic arbor and the morphology of neurons projecting to a specific region (Laramée et al., 2013a).

### Cellular activity

Certain areas can also demarcated based on their metabolic activity, directly linked to cytochrome oxidase activity in the cell. Therefore, a simple staining technique can be used to quantitatively examine cellular activity in different visual cortices, and compartments within them (Wong-Riley, 1979). This technique is routinely used to locate the representation of the whiskers in the “barrel fields” of the rodent somatosensory cortex (e.g., Li et al., 2013) but is also able to demarcate V1 versus the extrastriate areas and is used in many species including mouse (Airey et al., 2005), cat (Wong-Riley, 1979), ferret (Innocenti et al., 2002), gray squirrel (Wong and Kaas, 2008), short-tailed possum (Wong and Kaas, 2009). In higher species, excluding rodents, cytochrome oxidase staining in V1 reveals characteristic blobs reflecting the columnar organization of visual inputs from the remaining eye in the context of a monoenucleation paradigm in the cat and the squirrel monkey (Wong-Riley, 1979; Carroll and Wong-Riley, 1984).

Visual areas can also be functionally identified by following transient changes in intracellular calcium levels associated with neuronal firing, revealed by synthetic indicators or genetically encoded calcium indicators (GECIs). GECIs are less invasive or damaging for the tissue than synthetic indicators and allow for chronic *in vivo* measurements however early generations produced inferior signals. New GCaMP variants have been engineered offering improved photostability and calcium sensitivity, including GCaMP3 which is capable of detecting transient calcium current with an amplitude linearly dependent on action potential number (Tian et al., 2009). Adeno-associated virus AAV2 coding for *GCaMP3* under the control of the *synapsin-1* promoter was recently used in combination with 2-photon imaging to decipher stimulus preferences in the visual cortex of awake behaving mice (Andermann et al., 2013). The authors reveal that the posterior medial (PM) and the anterior lateral (AL) areas present similar orientation selectivity but different spatial and temporal frequency; PM neurons respond best to slow-moving stimuli and AL neurons to fast-moving targets. These results were confirmed by flavoprotein fluorescence imaging (Tohmi et al., 2014). Two-photon calcium imaging is a cutting-edge approach but requires pre-existing knowledge of the cortical map to determine calcium indicator injection sites, however it allows systematic functional mapping in small animal models, comparably to electrophysiology.

Neuronal activity also triggers the expression of immediate early genes (IEG), such as *zif268* and *cFos* (Figure 1D). IEGs are activated transiently and rapidly in response to cellular activity and monitoring their expression by immunostaining or RNA *in situ* hybridization. This can efficiently label visual territories in the vervet monkey, cat, mouse and the rat (Chaudhuri et al., 1995; Lyford et al., 1995; Zangenehpour and Chaudhuri, 2002). To achieve optimal signal-to-noise ratio, experimental animals are first subjected to a period of dark adaptation, to reduce basal activity level to a minimum followed by a brief, intense light stimulation period, immediately prior to perfusion. This technique is particularly effective to determine ocular dominance in mouse V1 by specifically blocking the input from one eye (e.g., eyelid closure or enucleation) during the phase of light stimulation (Van der Gucht et al., 2007). IEGs are also advantageous to study neuroplasticity, especially during development and have been utilized for this in the mouse (Van Brussel et al., 2011; Nys et al., 2014).

The markers presented above are extremely effective at demarcating V1 and associated subcompartments, in non-primate species, but they prove limited in demarcating higher order areas. Higher order areas do not exhibit sharp cytoarchitectural differences, especially in the rodents, however their cellular composition varies greatly which can be captured with cell-type specific markers.

### Cell-specific markers

The most frequently used cell-specific protein for mapping visual cortical areas in numerous species has been the nonphosphorylated isoform of high molecular weight neurofilament (NNF). The protein is an intermediate filament, a major component of the neuronal cytoskeleton, and development of a specific antibody—SMI-32 (Sternberger and Sternberger, 1983), led to an explosion in the capacity to further demarcate the extrastriate visual cortex of a number of species. NNF is specifically expressed in the basal and apical dendrites of excitatory cortical neurons in layers 2, 3, 5 and 6 and reveals specific details of the cell morphology (Figure 1C). Immunolabeling against NNF reveals the morphology of the dendritic tree, which varies dramatically across visual areas and across cortical layers. NNF expression profile has been established in a large number of non-primate species, including cat, ferret, mouse, rat (van der Gucht et al., 2001; Van der Gucht et al., 2007; Sia and Bourne, 2008; Homman-Ludiye et al., 2010) and is remarkably conserved across equivalent visual areas leading to a clearer understanding of the evolution of species within an order (e.g., in the cat and the ferret visual cortex (van der Gucht et al., 2001; Homman-Ludiye et al., 2010)). In the visual cortex, NNF protein content directly correlates with the conduction speed of an axon (Hoffman et al., 1987; Lawson and Waddell, 1991) and primary sensory cortical areas across modalities exhibit the highest concentration of NNF expression. High levels of NNF protein are found in fast-conducting fibers and cortical areas belonging to the dorsal visual processing stream (Gutierrez et al., 1995; Chaudhuri et al., 1996; Bourne and Rosa, 2003). This property, initially demonstrated in primate species, is conserved in carnivores (van der Gucht et al., 2001; Homman-Ludiye et al., 2010) and rodents (Van der Gucht et al., 2007). Furthermore, NNF can be used to demonstrate the maturation of visual cortical areas, as it is only expressed in structurally mature neurons. This feature has been used to map the development of areas in the visual cortex, primarily in the nonhuman primate (Bourne et al., 2005; Bourne and Rosa, 2006), demonstrating that the MT is a V1 (Bourne and Rosa, 2006; Bourne et al., 2007). Unfortunately, this property of NNF has not been taken advantage of in other species.

Visual cortical areas also exhibit a distinctive expression profile of chondroitin sulphate proteoglycan (CSPG). CSPGs constitute the extracellular matrix of most neurons, they are highly heterogeneous (Matthews et al., 2002) and are first detected at late developmental stages where they are believed to contribute to the transition to an extracellular environment non-permissive to migration (Celio et al., 1998). The antibody clone Cat-301 can detect the CSPGs and therefore, labels the cell body and proximal dendrites (McKay and Hockfield, 1982; Zaremba et al., 1989) around synapses but not the synaptic cleft (McKay and Hockfield, 1982; Hockfield et al., 1990). In the cat and old world monkey neocortex, Cat-301 labeling is restricted to layers 3 and 5 in most areas and, additionally layers 4 and 6 in primary sensory areas (Hendry et al., 1988), with a high degree of variation across association cortex areas which allows for demarcating areal borders. Numerous visual cortices of non-primate species can be demarcated utilizing the Cat-301 antibody, such as the cat (Hendry et al., 1988) and the ferret (Homman-Ludiye et al., 2010). In the visual cortex, similarly to NNF, Cat-301 is preferentially associated with dorsal stream areas in nonhuman primates (Hendry et al., 1988; Hof et al., 1995).

In addition to markers such as NNF and Cat-301, visual areas can also be defined according to the distribution of GABAergic interneurons subtypes. In particular, interneurons expressing the calcium-binding proteins Calbindin-D28k (Cb) and Parvalbumin (Pv) reveal complementary subpopulations of GABAergic interneurons differentially distributed across visual cortical areas (Figures 1E,F). Their developmental expression profile has been well documented in the primate visual cortex, revealing an early onset of Cb during corticogenesis and a later upregulation of Pv, around birth in layers 4–6 (Hendrickson et al., 1991) but has yet to be translated into non-primate species. The early expression of Cb is very dynamic in terms of amount, laminar distribution and cell types labeled. After birth and in the adult brain, Cb expression stabilizes in the supraganular layers, whereas Pv expression tends to be associated with cells in the infragranular layers. The interneuron subpopulations do not overlap in the cat visual cortex (Demeulemeester et al., 1988, 1991) but this is less clear in rodents. The role of these molecules remains poorly understood beyond calcium buffering but it has been suggested that Cb is associated with the formation of synapses and Pv, with the onset of functional activation during cortical maturation (Hendrickson et al., 1991). Cb and Pv have been extensively used to map the neocortex of numerous non-primate species, including the gray squirrel (Wong and Kaas, 2008) and marsupials such as the echidna, opossum, dunnart, antechinus and phascogale (Hassiotis et al., 2004; Ashwell et al., 2008; Wong and Kaas, 2009) in combination with myelin and cytoarchitectural markers. In the opossum, which also possesses a relatively small V1, the expression of Pv is restricted to V1 and does not extend into adjacent areas, while Cb is almost absent from the brain (Wong and Kaas, 2009). However, the highly visual gray squirrel exhibits a high level of Pv and Cb expression across most of the neocortex (Wong and Kaas, 2008), and Pv is very weakly expressed in the limited visual cortex of the echidna (Hassiotis et al., 2004). The comparison of these maps confirms that the expression of the calcium binding proteins Cb and Pv is highly dependent on the activity of a visual area and is upregulated in the visual cortex of species relying on visual input to interact with their environment. Their expression is therefore relative to their function in buffering calcium within the cell.

The 36-amino acid Neuropeptide Y (NPY) is involved in synaptic transmission, cerebral blood flow regulation, and inhibition of neuronal excitability (Raghanti et al., 2013) which is predominantly expressed by GABAergic interneurons. NPY+ interneurons exhibit bipolar, bitufted or multipolar morphology and are more concentrated in layers 2, 3 and 6. In the macaque, NPY+ neurons exhibit an area-specific distribution (Kuljis and Rakic, 1989a) with a high inter-animal variability. In the cat, NPY immunopositive neurons are homogeneously distributed across striate and extrastriate areas 17, 18 and 19, accumulating in layers 5 and 6 where they contribute for 0.2% and 1.5% of the total neuronal population, respectively (Demeulemeester et al., 1988). Whilst no difference in NPY distribution was originally detected in the rat visual cortex (Allen et al., 1983), a more recent analysis of *NPY* mRNA distribution established a two-fold expression increase in V2 compared to V1 at postnatal day 21 (Obst and Wahle, 1995). Visual activity is required to maintain the phenotype of supragranular NPY+ neurons in the rat V1 (Obst et al., 1998). The non-uniform laminar distribution of NPY in axons across areas is less variable between animals than the density of NPY containing somata (Kuljis and Rakic, 1989a,b). Therefore, the relative density of NPY-containing axons can be used as an additional chemoarchitectonic criterion to demarcate and characterize cortical areas. This method can be extended to multiple non-primate species as comparable pattern and density variations of NPY+ neurons have been observed in dolphin, manatee, walrus, seal, elephant (Butti et al., 2011), and species belonging to xenarthra superorder (tree sloths and armadillos) and afrotheria clade (hyraxes and elephants) (Sherwood et al., 2009). In these species, NPY distribution is concentrated again in layers 5 and 6 and the underlying white matter (Butti et al., 2011).

Since cortical areas are classically defined by anatomical, and functional criteria (Kaas, 1995), maps based on a single criterion can be inaccurate making it difficult to reconcile different studies. An example of this can be observed in the demarcation of the mouse visual cortex where different criteria have resulted in different maps (Van der Gucht et al., 2007; Wang and Burkhalter, 2007). By combining the markers and methods we presented above, investigators have been extremely successful in mapping the visual cortex of a variety of species who have a differing reliance on vision, which allows us the opportunity to retrace the evolution of the visual cortex. Achieving this goal requires developing a consensus on the visual cortical map of a particular species, but also across species, and what specific criteria are necessary to define each cortical area. This is of particular importance as advance in technologies provides a great opportunity to identify new areas.

## Evolution and homology of visual cortical areas

The fissure pattern and the overall size of the brain of long extinct species can be deduced from endocasts of their fossilized skulls but being soft tissue, the brain is not preserved making it impossible to establish how the organization of cortical fields has been remodeled across evolution. To retrace the steps that have led to the variety of modern cortical maps, including the complex primate visual cortex, investigators have devised a comparative approach under the principle that the different levels of visual cortex complexity displayed by current species illustrate different steps along the evolutionary path (for review, see Krubitzer and Hunt, 2007). By comparing cortical maps across mammalian orders, one can determine which features are homologous, and therefore inherited from a common ancestor. For example, it was believed that the organization of visual areas into a dorsal stream, specialized in interpreting information relating to the position of an object, and a ventral stream dedicated to object recognition (Mishkin and Ungerleider, 1982; Ungerleider and Haxby, 1994) was exclusively present in primate species. The recent discovery of two processing streams in the mouse visual cortex (Wang et al., 2012) suggests that this trait is homologous in rodents and primates and probably appeared early on in evolution. The diversity of environments colonized by mammals imparts valuable information regarding the stability of the visual system and it is therefore crucial to investigate the largest variety of species possible, facilitated by the use of non-electrophysiological approaches. Some features are actively defended against change across niches such as the specification of V1 and V2 areas, which are both present in the mole rat despite being subterranean and virtually blind (Matsunaga et al., 2013). Alternatively, other characteristics have appeared in a specific lineage as an adaptation to modifications of the ecological niche (Bullock, 1984).

Two important aspects to consider when comparing the visual cortical map of separate species are the brain size and the ecological niche (Finlay et al., 2014). Originally, mammals were nocturnal (Hall et al., 2012) and in every order today, we find nocturnal species possessing a smaller brain and a rudimentary visual system compared to the large-brained diurnal species (Ross, 2000). But it is now evident that a larger brain is not equivalent to a more complex brain (Manger, 2005). The recent comparative analysis of the cat and the ferret visual cortex, two carnivores that diverged 5 million years ago (Bininda-Emonds et al., 1999), revealed the same number of visual areas despite the cat brain being 6-fold larger (30 g versus 5 g) (van der Gucht et al., 2001; Manger et al., 2005; Homman-Ludiye et al., 2010). Similarly, the highly visual marmoset monkey (*Callithrix jacchus*) visual cortex comprises more areas and enhanced visual ability but a comparatively smaller brain than the cat. Therefore, the evolutionary expansion of the neocortical surface (Rakic et al., 2009) does not directly correlate with the addition of visual areas in higher species (Kaas, 1997). It has been proposed that the complexity of neural system, corresponding to the number of cortical divisions and subcortical nuclei, increases with the establishment of a new mammalian order (Manger, 2005).

Analysis of the squirrel visual system, a highly visual diurnal arboreal rodent who shares similar ecological constrains with primates, demonstrates more similitude with mammals which are more closely related to primates, such as the tree shrew, than the mouse (Paolini and Sereno, 1998; Campi and Krubitzer, 2010). This includes the presence of a five-layered laminated LGN compared to the three-layered rat LGN (Kaas et al., 1972; Montero, 1993) and a pulvinar nucleus (Baldwin et al., 2011), a thalamic nucleus absent in most rodents. This observation suggests that the ecological niche exerts more pressure than the boundaries of a phylogenetic group (Campi and Krubitzer, 2010). Some features, including the presence of a complex pulvinar nucleus, reflect adaptive changes or specialization at the level of individual species, taxon or niche (Finlay et al., 2014). Suggestions that the rodent lateral posterior nucleus (LPN) is the equivalent of the pulvinar nucleus (see Lyon et al., 2003a,b; Kaas and Lyon, 2007) are supported by a recent study demonstrating the importance of the superior colliculus-LPN-higher visual areas pathway and that connections with different higher order areas are segregated to specific discrete domains in the LPN (Tohmi et al., 2014). However this organization does not compare to the functional parcellation and exquisite cytoarchitecture characteristic of the primate pulvinars nucleus. The investigation of the developmental origin of LPN and pulvinar nucleus in rodents and primates will certainly help resolve this ambiguity.

Although the suggestion is that a larger brain does not correlate with a more complex brain (Manger, 2005), the addition of new areas is certainly concomitant with the expansion of the cortical surface, however it is unclear if one event prompted the other. The generation of a larger neocortical sheet occurred through modifications of the cell cycle and division mode of cortical progenitors, including expansion of the progenitor pool by increasing cell cycle re-entry. Forcing cell cycle re-entry by upregulating the cell cycle regulators Cdk4 and CyclinD1 in the mouse appears to recapitulate the evolutionary expansion of the cortical surface without thickening of the cortical layers (Nonaka-Kinoshita et al., 2013). Indeed, the human neocortex is 1000 times larger than that of the mouse but only twice as thick (Blinkov and Glezer, 1968; Rakic, 1995). A study in the macaque suggested that differences in cell cycle regulation could also be observed at the level of a single area, revealing higher proliferation rates in V1 compared to V2 (Lukaszewicz et al., 2005). Analysis of the ferret, sheep, cat and mouse neocortex confirmed that mitotic cells do not distribute evenly during development, however this study demonstrated that fast cycling progenitors accumulate in regions undergoing the greatest tangential expansion, corresponding to presumptive gyri (Reillo et al., 2011). It is therefore possible that the more intense proliferation in the macaque V1 compared to V2 is a topologic feature independent of the area identity or function, and reflects the lateral expansion of the primary visual cortex leading to the formation and folding of the calcarine sulcus. The folding of the neocortical sheet is an important feature in the elaboration of a larger neocortex (Zilles et al., 2013) in order to maintain a reasonable head to body size ratio. The pattern of gyri and sulci exhibits inter-individual variation but is largely conserved within a species suggesting a genetic control. Local regulation of *Trnp1* (Stahl et al., 2013) and *GPR56* (Bae et al., 2014) in the mouse induces the formation of folds in the smooth rodent brain, illustrating the importance of multispecies approaches.

While we have garnered a better grasp on the principles of the evolution of the visual cortex and the mechanisms underlying the expansion of the cortical surface, the driving forces leading to the emergence of new visual areas with novel function and an original identity remain largely unknown. Elucidating the developmental regulation controlling the patterning of the neocortex and visual areas identity specification will undoubtedly provide answers regarding the evolution of the visual cortex, including the advantage of adding more areas instead of developing new functions in pre-existing ones.

## Genetic specification of neocortical domains

**Figure 2:**
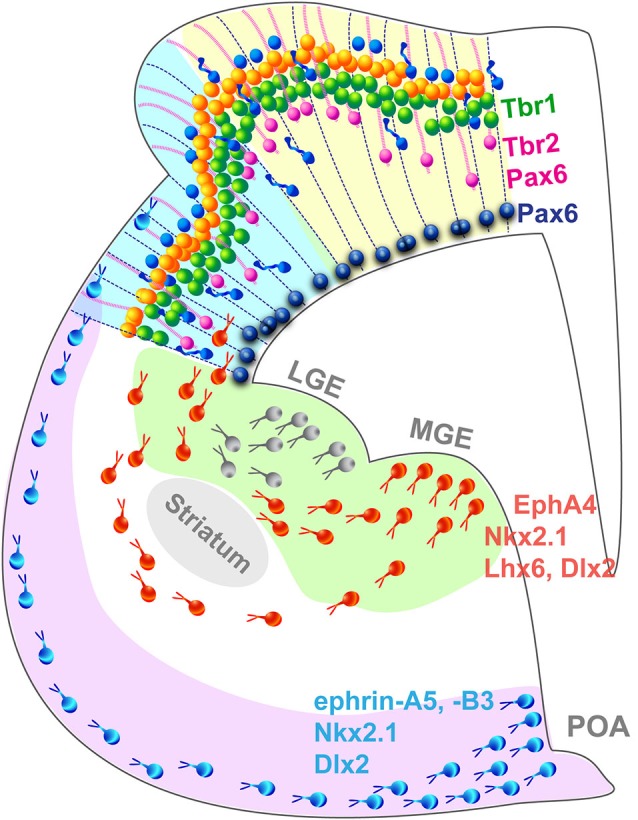
**Summary schematic representing the principal cell populations and mechanisms involved in the formation of a complex gyrated neocortex**. Pax6+ radial glia cells (blue) are attached to the ventricular surface and extend parallel processes to the pial surface of the cortex. In higher mammals, an additional population of Pax6+/Tbr2+ progenitors (pink) attached exclusively to the pial surface contribute to the radial expansion of the neocortex including the formation of folds. Newborn neurons migrate radially in an inside-out pattern. Interneurons migrate tangentially from subcortical origins along a superficial and a deep migratory stream, guided by a combination of attracting and repulsive cues. *LGE*, lateral ganglionic eminence; *MGE*, medial ganglionic eminence; *POA*, preoptic area.

Cortical layers originate from the proliferation of progenitor cells (PCs) in the neurogenic compartment of the developing neocortex lining the surface of the ventricle. PCs in the ventricular and subventricular zones (VZ; SVZ) divide symmetrically to generate two progenitor daughter cells to amplify the pool of PCs and expand the ventricular surface laterally (Figure 2). Alternatively, asymmetrical PCs division give rise to a single neuron and a PC or an intermediate progenitor cells (IPC) and a PC. IPCs are the main source of cortical neurons, they reside in the SVZ where they divide to produce two neurons or two IPCs (Haubensak et al., 2004; Kawaguchi et al., 2008; Pontious et al., 2008; Kowalczyk et al., 2009). Gyrencephalic species exhibit an enlarged SVZ, divided in an inner and outer compartments, ISVZ and OSVZ respectively, which is absent in non-gyrencephalic rodents (Smart et al., 2002; Lukaszewicz et al., 2005; Zecevic et al., 2005; Dehay and Kennedy, 2007; Bayatti et al., 2008; Martínez-Cerdeño et al., 2012). In addition to IPCs, the OSVZ contains radial glia cells similar to those found in the VZ but they lack an apical process attaching them to the VZ, and possess a single basal process along which the cell body moves during the cell cycle (Hansen et al., 2010; Reillo et al., 2011; Shitamukai et al., 2011; Gertz et al., 2014). OSVZ radial glia cells (oRGC) self-renew and generate neurons directly, participating to the gyrification of larger brains but are also found in limited amount in the mouse cortex (Wang et al., 2011). The newborn neurons then migrate along a radial process in an inside-out fashion to form the cortical layers (Kriegstein and Noctor, 2004; Molyneaux et al., 2007) where they mature and establish short-range connections with neighboring cells and long-range connections with other areas or subcortical regions (for review see Marín and Rubenstein, 2003).

GABAergic interneurons populate the neocortex through a different mode (Figure 2): most are born in subcortical domains, the ganglionic eminences (GE) and the pre-optic area (POA; Gelman et al., 2009; Zimmer et al., 2011; Sultan et al., 2013) and migrate tangentially until they reach the neocortex and then switch to a radial mode to integrate into the cortical network (Nery et al., 2002; Ang et al., 2003; Marín and Rubenstein, 2003). This migration mode has been demonstrated in the mouse, however studies suggest that in nonhuman primates, additional waves of interneurons are generated locally in the neocortex and migrate radially along a similar route to that followed by pyramidal neurons (Letinic et al., 2002; Rakic, 2002). The controversial hypothesis of locally born neocortical interneuron populations is appealing because it provides a mechanism by which interneurons might have adjusted to the increasing distance between the traditional interneurogenic sites and the neocortex during the evolutionary expansion of the brain. Recent evidence arguing against a neocortical pool of interneuron progenitors in the embryonic macaque and human (Ma et al., 2013) endeavored to close the debate, however the study focused on early stages of neocorticogenesis and did not analyze later waves of neocortical interneurons which most likely originate locally as they are born in a brain of larger dimension. In addition, the authors analyzed the interneurons emerging from the GE exclusively, without taking into account the contribution of the POA recently demonstrated as a source of interneurons in the mouse (Gelman et al., 2009; Zimmer et al., 2011). Considering the substantial increase of the proportion of interneuron in the neocortex during evolution, which constitute 15% of the total neuronal population in the mouse neocortex compared to 24–30% in primates (for review see Rudy et al., 2011), it is plausible that sites of interneuron genesis must have increased not disappeared, supporting the hypothesis of neocortical interneuron progenitors. Alternative intermediate models, such at the ferret or the cat, with a complex brain likely to comprise a mixed interneuronal population similar to the primate but a simpler visual cortex, will without a doubt play an important role in resolving the debate. Encouragingly, interneuron migratory routes are beginning to be characterized in the developing ferret brain, in the context of cortical dysplasia (Poluch et al., 2008; Abbah and Juliano, 2013).

Although the generation of cortical neurons and interneurons is well characterized, progress on area patterning has been slow. Two opposing models of cortical patterning were originally proposed to explain the phenomenon. The “tabula rasa” hypothesis states that the neocortex begins as a blank slate and is patterned solely by the innervation of thalamic afferents (O’Leary, 1989), while the “protomap” hypothesis argues that cortical identity is predetermined, already present in PCs in the neurogenic zones and subsequently transferred to the progeny (Rakic, 1988). The current theory suggests that in fact, both theories are in play (for review O’Leary et al., 2007). Areas initially acquire their identity through a combination of intrinsic molecular programs and their borders are later refined via signals carried by the thalamic axons, who also provide the cortical domains’ functional identity. The precedence of intrinsic over extrinsic signals in conferring area position suggests that new areas could arise from a modification of the gene expression profile present in a particular cortical region at a given time. In order to identify the modifications that have led to more areas, one must first understand the regulatory events in a simple brain with fewer cortical areas, such as the mouse, which also affords the potential for manipulating gene expression at the cellular level.

The first step of cortical patterning is achieved through the graded expression of transcription factors and homeobox genes along the axes of the brain to define domains with a unique combination. In the embryonic mouse brain, the transcription factor Paired Box 6 (Pax6) is expressed in a high anterior/low posterior and high lateral/low medial gradient (Walther and Gruss, 1991; Stoykova and Gruss, 1994). The transcription factor Emx2 is expressed in an opposing gradient, with low anterior/high posterior and low lateral/high medial gradients (Gulisano et al., 1996; Mallamaci et al., 1998). Removing either transcription factor (TF) dramatically affects the organization of cortical areas. In *Emx2* knock out (KO) mice, the anterior territories, including the somatosensory cortex and the motor cortex, expand and take over more posterior domains, leading to a reduction of the visual cortex. The situation is reverted in *Pax6* KO where the visual cortex expands rostrally with detrimental effects on anterior areas (Bishop et al., 2000). This pivotal finding demonstrates that *Emx2* is capable of repressing the “anterior identity” and specify visual identity in the immature cortical plate (Bishop et al., 2000). Similarly, the transcription factor *COUP-TFI* is upregulated in the caudoventral portion of the neocortex (Liu et al., 2000) and promotes caudal area identity including the visual areas (Armentano et al., 2007), in part by downregulating *Pax6* expression along the dorsoventral axis and blocking the “anterior identity” (Faedo et al., 2008).

Gradients of transcription factors across the embryonic neocortex are established by diffusible morphogens, including BMPs, Wnts and Fgfs. Fgf8 and Fgf17 to a lesser extent, are secreted by the anterior neural ridge (ANR) and contribute to promoting anterior identity by negatively regulating the expression of *Emx2* and *COUP-TF1* (Garel et al., 2003; Grove and Fukuchi-Shimogori, 2003; Cholfin and Rubenstein, 2007). Fgf8 upregulates the expression of the zinc-finger transcription factor *Sp8* (O’Leary and Sahara, 2008) which inhibits Emx2 by direct interaction (Zembrzycki et al., 2007) therefore Sp8 contributes to anterior territories specification and represses visual identity (Borello et al., 2014). Using genetic models of loss and gain of function, target genes regulated by Pax6 are slowly being identified (Quinn et al., 2007), shedding light on how the gradual regional identity is propagated from PCs in the neurogenic zones to mature cortical neurons in order to establish areal boundaries. Recent evidence suggests that the positional identity is maintained across the successive differentiation stage and zones by a specific cascade of transcription factors. Tbr2 expression in IPCs, directly activated by Pax6 (Sansom et al., 2009), is detected in a high rostral/low caudal gradient across the SVZ (Bulfone et al., 1999; Krüger and Braun, 2002; Bedogni et al., 2010) reminiscent of Pax6 expression profile in the VZ. The conditional loss of *Tbr2* (also known as *Eomes*) in the mouse neocortex at embryonic day 11 (E11) leads to the downregulation of rostral markers in the CP at E14.5 (Arnold et al., 2008; Sessa et al., 2008; Elsen et al., 2013) and perturbation of the anterior regional identity leading to disorganized somatosensory “barrel fields” (Elsen et al., 2013). Therefore, in addition to promoting IPC genesis, Tbr2 participates to cortical patterning and relays Pax6 positional information (Elsen et al., 2013) in neurons entering the cortical plate by activating the expression of the transcription factor Tbr1 (Englund et al., 2005). *Tbr1* expression is reduced in *Tbr2* conditional knockout mice (Elsen et al., 2013), and anterior patterning is disorganized in *Tbr1* mutants (Arnold et al., 2008; Sessa et al., 2008), suggesting that *Tbr1* carries the rostral identity in the cortical neurons. A similar genetic sequence for the specification of the visual cortex has not yet been identified, however the transcription factor *Bhlhb5* (also known as *Bhlhe22*) is expressed in a profile similar to that of *Emx2* and is thought to regulate the posterior identity acquisition in cortical neurons (Joshi et al., 2008). *Bhlhb5* is therefore a privileged candidate for visual cortex patterning. The patterning of subcompartments within visual areas also comprises an intrinsic component. Researchers investigating the development of ocular dominance columns in the cat visual cortex recently identified the heat shock protein 90 alpha (Hsp90α) to be specifically associated with ipsilateral connections. They reveal that clusters of cells expressing Hsp90α form in the visual cortex 2 weeks before the development of the columns, setting the initial pattern for optical dominance columns (Tomita et al., 2013). The absence of columns in the rodent precludes this research to be completed.

**Figure 3:**
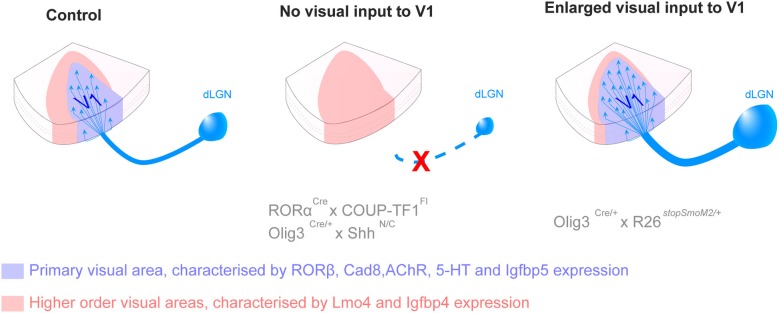
**Thalamic connections contribute to the acquisition of primary versus secondary area identity postnatally**. Loss of inputs from the dorsal lateral geniculate nucleus (dLGN), by genetic deletion, leads to the absence of the primary visual area (V1, blue) and the corresponding territory adopts a secondary area (red) identity. Opposite changes are observed in presence of additional dLGN inputs, with an enlargement of V1 and a reduction of secondary domain. The size of the LPN, the thalamic nucleus projecting to higher order visual areas, varies in a similar manner as the size of the higher visual area, suggesting feed-back regulation. The mechanisms by which thalamic axons influence the fate of cortical neurons in not yet understood (*For more details see Chou et al., 2013; Vue et al., 2013)*.

Candidate genes responsible for cortical patterning and visual area specification have mainly been identified in the mouse and it is not known yet to what degree their roles can be translated in higher species. Pax6 patterning function resides in its gradual distribution across the anteroposterior axis during development, demonstrated in the mouse. However, Pax6 is consistently expressed in oRGC throughout the OSVZ of gyrencephalic species (Reillo et al., 2011), suggesting that Pax6 might have lost its patterning properties during neocortical expansion. Quantitative studies comparing gene expression level in various region of the brain, including microarray and quantitative real time polymerase reaction, in gyrencephalic species are needed to validate area specification pathways identified in the mouse. The specification of discrete visual areas is genetically controlled but the functional identity is carried by axons emerging from the visual relay nuclei of the thalamus and projecting to layer 4 in the neocortex. Recently in the mouse, new genetic models that specifically obliterate input to the neocortex, combined with molecular demarcation of area borders, have enabled the elucidation of the role of cortical afferents in area specification. By specifically deleting the expression of the transcription factor *COUP-TFI* in the dorsal lateral geniculate nucleus (dLGN), researchers have demonstrated that geniculocortical inputs drive the genetic distinction between primary and higher-order areas (Chou et al., 2013; Vue et al., 2013). Vue and colleagues also reveal that the surface of V1 in the mouse varies with the modification of the size of the LGN (Vue et al., 2013). These results are recapitulated in Figure 3.

The refinement of gene transfer techniques, in particular* in utero* electroporation, can help to bridge the gap with other species. This technique allows for gene transfer in restricted portions of an epithelium by application of a series of electric pulses (Saito and Nakatsuji, 2001). Groups around the world are taking advantage of this technique to characterize the genes involved in visual cortex patterning in species more dependent on vision, like the ferret, therefore offering a more relevant substrate (Kawasaki et al., 2012). Undoubtedly, the combinatorial distribution of transcription factors has increased with the addition of new visual areas by modifying their expression domain and/or the timing of their expression. We are getting closer to breaking the code underlying the specification of a large number of areas, in particular with the development of microarray in a large number of species and next generation sequencing, which identifies all the gene products present in a given region, including non-coding regulating sequences (Ayoub et al., 2011; Belgard et al., 2011; Bernard et al., 2012; Oeschger et al., 2012). However, it is important to also decipher how these genes affect individual cell behavior, which ultimately leads to the formation of characteristic areal boundaries and the specific function of areas within a specific domain, such as the visual cortex.

## Molecular control of visual cortical arealisation

The transcription factors discussed above exhibit graded expression throughout the developing cortical compartments and it is not known how their “blurry” limits are translated into the sharp boundaries characteristic of the visual areas in the mature neocortex. Spatiotemporal mapping of the visual cortex in different species demonstrates a combinatorial expression of guidance molecules, dynamically regulated during development. Each subtype of guidance molecule defines a permissive or repulsive environment for subsets of cortical neurons. Remarkably, during development the expression of guidance molecules demonstrates sharp boundaries, often matching the borders of the putative area. In addition, guidance cues distributed in an area-specific profile also contribute to guiding intracortical connections as well as connections between the neocortex and subcortical regions, contributing to the specification of an areas functional identity.

Guidance cues are traditionally divided into two categories: secreted molecules that diffuse in the extracellular space and membrane-bound molecules attached to the cell surface and requiring close proximity between the two interacting cells. Interaction between the ligand and its specific receptor(s) expressed on the surface of the target cell, elicits a cascade of intracellular reactions leading to the reorganization of the cytoskeleton. Signaling pathways promoting microtubule polymerization attract responsive cells towards the source of ligand. Conversely, collapse of the microtubule scaffold results in repulsion and the target cell moves away from the source of guidance molecule. The migratory response to a particular guidance molecule is highly influenced by the environment and the combination of receptors and co-receptors expressed on the target cell, thus the same guidance molecule can be both attractive and repulsive (Lehigh et al., 2013).

### Eph/Ephrins

The first evidence of the implication of guidance molecules in area formation illustrated the selective expression of *EphA* family members in the developing macaque neocortex (Donoghue and Rakic, 1999). Eph receptors (A and B) belong to the large family of tyrosine-kinase receptors activated by cell surface ligands, the *ephrins*. Ephrin-As are attached to the membrane via a glycosyl phosphatidylinositol (GPI) anchor, while the ephrin-Bs are transmembrane (Flenniken et al., 1996; Brückner and Klein, 1998). Activation of the receptor often results in repulsion of the cell (Gale and Yancopoulos, 1997; Hattori et al., 2000). The receptor-ligand interaction is also capable of eliciting a response in the ligand-bearing cell, a phenomenon known as reverse signaling (Holland et al., 1996; Gale and Yancopoulos, 1997). Eph/ephrin signaling is involved in many aspects of development, including blood vessels and topographic organization of retinal projections; animals with defective Eph/ephrin signaling usually exhibit aberrant connectivity (Friedman and O’Leary, 1996; Gale and Yancopoulos, 1997; Flanagan and Vanderhaeghen, 1998; Frisén et al., 1998; Feng et al., 2000; Helmbacher et al., 2000). The *Eph/ephrin* RNA expression profile in the embryonic primate neocortex reveals an area-specific patterning, providing the first evidence of the early specification of presumptive functional domains (Donoghue and Rakic, 1999). Similar analysis in the mouse demonstrates that *EphA6* expression is restricted to the posterior pole of the developing neocortex, suggesting a selective guidance mechanism for excitatory neurons into the future visual cortex (Yun et al., 2003). The specific expression of *EphA6* in the presumptive visual cortex is independent of thalamic inputs as it is not affected in *Mash1* KO animals, which fail to develop inputs from the LGN (Nakagawa et al., 1999; Yun et al., 2003). *EphA7* and *ephrin-A5* are mutually exclusive and absent from the presumptive visual cortex with *EphA7* restricted to the anterior end of the developing mouse neocortex and *ephrin-A5* delineating a specific domain in the middle of the A-P axis (Yun et al., 2003). In *Mash1*−/−, *EphA7* expression domain expands posteriorly and overlaps with *ephrin-*A5 to define a new region (Yun et al., 2003). In addition to steering excitatory neurons to appropriate neocortical areas, activation of EphA7 by ephrin-A5 controls brain size by regulating apoptosis of neural progenitors (Depaepe et al., 2005). The discrete ephrin-A5 expression profile suggests that EphA7/ephrin-A5 dependant apoptosis takes place in an area specific manner, providing an additional regulatory mechanism for area specification. Ephrin-B1 also contributes to excitatory neuron migration by restricting their lateral migration and maintaining the columnar organization of the progeny of a single progenitor cell (Dimidschstein et al., 2013). Unfortunately, this study does not take into account the arealisation of the neocortex. We can hypothesize differential ephrin-B1 regulation at the level of the border between two areas, where the lateral spread of cortical neurons would be more strictly controlled to segregate different populations compared to neurons within an area. In addition to its roles during development, Ephrin-B1 expression is sustained in postnatal and adult marmoset monkey visual cortex (*Callithrix jacchus*, Teo et al., 2012) suggesting a role in maintenance of connectivity and ongoing neuroplasticity which need to be further investigated and confirmed in other species.

We recently described EphA4 expression profile during development, in the visual cortex of the marmoset monkey (Goldshmit et al., 2014), revealing major differences with the mouse, including robust expression of EphA4 on glial cells in the adult, which normally disappears in rodents at the end of neurogenesis. This finding implies that EphA4 bears additional function in the primate visual cortex compared to the mouse. Although these roles have yet to be characterized, it will be important to analyze the expression of Eph/ephrin family members in alternative species to identify potential modifications and associate with the evolution of the neocortex. Despite the prevalence of Eph/ephrin in corticogenesis, few studies have been performed in non-primate species other than the mouse, except a functional study of the ferret retinothalamic projections (Huberman et al., 2005).

### Cadherins

Another example of guidance molecules implicated in arealisation is the family of adhesion molecules known as cadherins. Cadherins are glycoproteins expressed at the cell surface. These molecules engage in homophilic binding, to confer preferential adhesiveness to cell populations in a calcium-regulated manner (for review Redies and Takeichi, 1996; Takeichi, 2007). Cells expressing the same cadherin within a larger population will specifically aggregate with each other, and separate from cells expressing different cadherins. In addition to this qualitative segregation, cells expressing different levels of the same cadherin will also selectively associate, adding a quantitative variable (Steinberg and Takeichi, 1994). These properties make cadherins ideal candidates to sort cells across presumptive cortical areas. A thorough study of the expression profile of 10 cadherins in the ferret visual cortex, from early embryonic stage to adult, demonstrates a dynamic area-specific and layer-specific expression profile (Krishna et al., 2009). The authors identified several cadherins differentially expressed across the V1/V2 borders with cadherin20 and protocadherin10 selectively expressed in V1 and cadherin8 and -11 restricted to V2. Similarly to the ferret visual cortex, cadherins exhibit a graded and areal pattern in the mouse neocortex independent of thalamocortical inputs, confirming that the initial steps of arealisation are intrinsically regulated (Nakagawa et al., 1999). These observations in non-primate species have emphasized the crucial role of cadherins in controlling the selective migration of neurons into particular visual areas, prompting similar mapping studies in a primate model, the marmoset monkey (Matsunaga et al., 2013).

### Semaphorins

The Semaphorin family comprises secreted and membrane-bound proteins characterized by a semaphorin domain in N-terminal and an immunoglobulin loop. Members exposed at the cell surface contain an additional GPI anchor and an intracellular C-terminal domain (Kolodkin et al., 1993). They interact with Plexin and Neuropilin (Npn) receptors but are also capable of activating the vascular endothelial growth factor receptor (VEGFR) through the formation of a receptor-complex with Npn (Kolodkin et al., 1997). Semaphorins regulate the migration of a large range of cells, including interneurons (Zimmer et al., 2010; Hernández-Miranda et al., 2011) and endothelial cells (Kutschera et al., 2011). They also control axon pathfinding in the central and peripheral nervous systems (Deck et al., 2013). In the somatosensory system, Sema6A guides thalamic projections to the appropriate domain in the dorsal neocortex. In absence of *Sema6A*, the thalamocortical axons project to a more ventral region of the neocortex, leading to a disorganized barrel field (Little et al., 2009) and modification of cortical domain identity. The barrel field is characteristic of rodent models therefore it is not known if Sema6A patterning potential is conserved in other species.

Using a comparative approach, our laboratory demonstrated that the secreted Sema3A interacts with Npn1 to regulate area-specific neuron migration in the mouse and the marmoset monkey visual cortex (Homman-Ludiye and Bourne, 2013). Moreover, we suggest that Sema3A, despite being homogenously expressed throughout the developing mouse neocortex (Giger et al., 1998; Polleux et al., 2000), contributes to patterning posterior identity in the mouse through differential expression of its receptor *Npn1* in presumptive V1. The volume of V1 is reduced in *Sema3A* KO animals compensated by an expansion of anterior fields (Homman-Ludiye and Bourne, 2013).

With 20 members interacting with a wide variety of receptor-complex, semaphorins are great candidates to fine tune the migration of cortical neurons into appropriate cortical domain. Semaphorin activity can also be modulated by components of the extracellular matrix, including CSPG (Kantor et al., 2004) for which the maps illustrating arealised expression in the visual cortex are available in non-primate species (Homman-Ludiye et al., 2010; van der Gucht et al., 2001). Therefore it will be extremely useful to compare the profile of CSPG and semaphorins in a given species to postulate on the potential functional interactions between members of the two families.

## Conclusion

The visual cortex is one of the most studied neocortical domains, possibly because of the prominent role of vision in a number of species. A large part of vision research is undertaken in primate species however, the organization of the visual system is robust and well conserved across evolution allowing comparison of human gene expression with analogous data in the mouse (Lein et al., 2007). Even virtually blind subterranean species retain a visual cortex (Crish et al., 2006; Matsunaga et al., 2011). Therefore, non-primate species can be examined to understand the evolution and development of visual cortical areas, especially that of man, which are difficult to source, including embryonic tissue, and do not offer opportunity for genetic modifications like the mouse.

Utilizing a wide variety of species can help us understand the major traits of cortical arealisation, as they are expected to present the least cross-species differences and identify what makes the human visual cortex so unique. A recent study reveals that a heavy selection pressure weighs on genes responsible for setting the basic structure of the brain organization, whilst the genes exhibiting cross-species difference have non-widespread expression patterns. This demonstrates a reduced selection pressure on these genes or that distinct, subtle changes may be opted for in divergent species rather than global changes (Zeng et al., 2012). The results reported in this study support the use of mouse as a good model system for the understanding of human brain function while pointing out important differences in the cellular organization between mouse and human brains and the differential functions individual genes may play in each species.

In summary, it is evident that to understand the complexity of a specific sensory system, whether it is its evolution, development or function relies on the analyses of multiple species. While the principal focus has been on primates and rodents, evidence indicates the importance of other species in completing this story. The next decade will most likely focus on closing the gap in our knowledge through comparative studies employing molecular tools, which will not only assist in addressing questions of evolution and development but also in tackling specific neurological issues.

## Conflict of interest statement

The authors declare that the research was conducted in the absence of any commercial or financial relationships that could be construed as a potential conflict of interest.

## References

[B1] Abbah J. Juliano S. L. (2013). Altered migratory behavior of interneurons in a model of cortical dysplasia: the influence of elevated GABAA activity. Cereb. Cortex [Epub ahead of print]. 10.1093/cercor/bht073 23574639 PMC4128700

[B2] Airey D. C. Robbins A. I. Enzinger K. M. Wu F. Collins C. E. (2005). Variation in the cortical area map of C57BL/6J and DBA/2J inbred mice predicts strain identity. BMC Neurosci. 6:18 10.1186/1471-2202-6-18 15774010 PMC1079866

[B3] Allen Y. S. Adrian T. E. Allen J. M. Tatemoto K. Crow T. J. Bloom S. R. (1983). Neuropeptide Y distribution in the rat brain. Science 221, 877–879 10.1126/science.6136091 6136091

[B4] Andermann M. L. Gilfoy N. B. Goldey G. J. Sachdev R. N. S. Wölfel M. McCormick D. A. (2013). Chronic cellular imaging of entire cortical columns in awake mice using microprisms. Neuron 80, 900–913 10.1016/j.neuron.2013.07.052 24139817 PMC3840091

[B5] Ang E. S. Jr. Haydar T. F. Gluncic V. Rakic P. (2003). Four-dimensional migratory coordinates of GABAergic interneurons in the developing mouse cortex. J. Neurosci. 23, 5805–5815 12843285 10.1523/JNEUROSCI.23-13-05805.2003 PMC6741259

[B6] Armentano M. Chou S.-J. Tomassy G. S. Leingärtner A. O’Leary D. D. M. Studer M. (2007). COUP-TFI regulates the balance of cortical patterning between frontal/motor and sensory areas. Nat. Neurosci. 10, 1277–1286 10.1038/nn1958 17828260

[B7] Arnold S. J. Huang G.-J. Cheung A. F. P. Era T. Nishikawa S.-I. Bikoff E. K. (2008). The T-box transcription factor Eomes/Tbr2 regulates neurogenesis in the cortical subventricular zone. Genes Dev. 22, 2479–2484 10.1101/gad.475408 18794345 PMC2546697

[B8] Ashwell K. W. S. McAllan B. M. Mai J. K. Paxinos G. (2008). Cortical cyto- and chemoarchitecture in three small Australian marsupial carnivores: Sminthopsis macroura, Antechinus stuartii and Phascogale calura. Brain Behav. Evol. 72, 215–232 10.1159/000165101 18946209

[B9] Ayoub A. E. Oh S. Xie Y. Leng J. Cotney J. Dominguez M. H. (2011). Transcriptional programs in transient embryonic zones of the cerebral cortex defined by high-resolution mRNA sequencing. Proc. Natl. Acad. Sci. U S A 108, 14950–14955 10.1073/pnas.1112213108 21873192 PMC3169109

[B10] Bae B.-I. Tietjen I. Atabay K. D. Evrony G. D. Johnson M. B. Asare E. (2014). Evolutionarily dynamic alternative splicing of GPR56 regulates regional cerebral cortical patterning. Science 343, 764–768 10.1126/science.1244392 24531968 PMC4480613

[B11] Baldwin M. K. L. Wong P. Reed J. L. Kaas J. H. (2011). Superior colliculus connections with visual thalamus in gray squirrels (Sciurus carolinensis): evidence for four subdivisions within the pulvinar complex. J. Comp. Neurol. 519, 1071–1094 10.1002/cne.22552 21344403 PMC3686314

[B12] Bayatti N. Moss J. A. Sun L. Ambrose P. Ward J. F. H. Lindsay S. (2008). A molecular neuroanatomical study of the developing human neocortex from 8 to 17 postconceptional weeks revealing the early differentiation of the subplate and subventricular zone. Cereb. Cortex 18, 1536–1548 10.1093/cercor/bhm184 17965125 PMC2430151

[B13] Bedogni F. Hodge R. D. Elsen G. E. Nelson B. R. Daza R. A. M. Beyer R. P. (2010). Tbr1 regulates regional and laminar identity of postmitotic neurons in developing neocortex. Proc. Natl. Acad. Sci. U S A 107, 13129–13134 10.1073/pnas.1002285107 20615956 PMC2919950

[B14] Belgard T. G. Marques A. C. Oliver P. L. Abaan H. O. Sirey T. M. Hoerder-Suabedissen A. (2011). A transcriptomic atlas of mouse neocortical layers. Neuron 71, 605–616 10.1016/j.neuron.2011.06.039 21867878 PMC3163272

[B15] Bernard A. Lubbers L. S. Tanis K. Q. Luo R. Podtelezhnikov A. A. Finney E. M. (2012). Transcriptional architecture of the primate neocortex. Neuron 73, 1083–1099 10.1016/j.neuron.2012.03.002 22445337 PMC3628746

[B16] Bininda-Emonds O. R. Gittleman J. L. Purvis A. (1999). Building large trees by combining phylogenetic information: a complete phylogeny of the extant Carnivora (Mammalia). Biol. Rev. Camb. Philos. Soc. 74, 143–175 10.1017/s0006323199005307 10396181

[B17] Bishop K. M. Goudreau G. O’Leary D. D. (2000). Regulation of area identity in the mammalian neocortex by Emx2 and Pax6. Science 288, 344–349 10.1126/science.288.5464.344 10764649

[B18] Blinkov S. M. Glezer I. I. (1968). “Techniques of quantitative measurement of morphological structures of the central nervous system,” in The Human Brain in Figures and Tables: A Quantitative Handbook (New York: Basic Books. Inc., Publishers and Plenum Press), 4–110

[B19] Borello U. Madhavan M. Vilinsky I. Faedo A. Pierani A. Rubenstein J. (2014). Sp8 and COUP-TF1 reciprocally regulate patterning and Fgf signaling in cortical progenitors. Cereb. Cortex 24, 1409–1421 10.1093/cercor/bhs412 23307639 PMC4014177

[B20] Bourne J. A. Rosa M. G. P. (2003). Neurofilament protein expression in the geniculostriate pathway of a new world monkey (Callithrix jacchus). Exp. Brain Res. 150, 19–24 10.1007/s00221-003-1397-5 12698212

[B21] Bourne J. A. Rosa M. G. P. (2006). Hierarchical development of the primate visual cortex, as revealed by neurofilament immunoreactivity: early maturation of the middle temporal area (MT). Cereb. Cortex 16, 405–414 10.1093/cercor/bhi119 15944371

[B22] Bourne J. A. Warner C. E. Rosa M. G. P. (2005). Topographic and laminar maturation of striate cortex in early postnatal marmoset monkeys, as revealed by neurofilament immunohistochemistry. Cereb. Cortex 15, 740–748 10.1093/cercor/bhh175 15342427

[B23] Bourne J. A. Warner C. E. Upton D. J. Rosa M. G. P. (2007). Chemoarchitecture of the middle temporal visual area in the marmoset monkey (Callithrix jacchus): laminar distribution of calcium-binding proteins (calbindin, parvalbumin) and nonphosphorylated neurofilament. J. Comp. Neurol. 500, 832–849 10.1002/cne.21190 17177255

[B24] Brodmann K. (1909). Vergleichende Lokalisationlehre der Groβhirnrinde in Irhen Prinzipien Dargestellt auf Grund des Zellenbaues. Leipzig: Johann Ambrosius Barth

[B25] Brückner K. Klein R. (1998). Signaling by Eph receptors and their ephrin ligands. Curr. Opin. Neurobiol. 8, 375–382 10.1016/s0959-4388(98)80064-0 9687349

[B26] Bulfone A. Martinez S. Marigo V. Campanella M. Basile A. Quaderi N. (1999). Expression pattern of the Tbr2 (Eomesodermin) gene during mouse and chick brain development. Mech. Dev. 84, 133–138 10.1016/s0925-4773(99)00053-2 10473127

[B27] Bullock T. H. (1984). Comparative neuroscience holds promise for quiet revolutions. Science 225, 473–478 10.1126/science.6740319 6740319

[B28] Butti C. Raghanti M. A. Sherwood C. C. Hof P. R. (2011). The neocortex of cetaceans: cytoarchitecture and comparison with other aquatic and terrestrial species. Ann. N Y Acad. Sci. 1225, 47–58 10.1111/j.1749-6632.2011.05980.x 21534992

[B29] Campi K. L. Krubitzer L. (2010). Comparative studies of diurnal and nocturnal rodents: differences in lifestyle result in alterations in cortical field size and number. J. Comp. Neurol. 518, 4491–4512 10.1002/cne.22466 20886618 PMC3432265

[B30] Carroll E. W. Wong-Riley M. T. (1984). Quantitative light and electron microscopic analysis of cytochrome oxidase-rich zones in the striate cortex of the squirrel monkey. J. Comp. Neurol. 222, 1–17 10.1002/cne.902220102 6321561

[B31] Celio M. R. Spreafico R. De Biasi S. Vitellaro-Zuccarello L. (1998). Perineuronal nets: past and present. Trends Neurosci. 21, 510–515 10.1016/s0166-2236(98)01298-3 9881847

[B32] Charbonneau V. Laramée M.-E. Boucher V. Bronchti G. Boire D. (2012). Cortical and subcortical projections to primary visual cortex in anophthalmic, enucleated and sighted mice. Eur. J. Neurosci. 36, 2949–2963 10.1111/j.1460-9568.2012.08215.x 22780435

[B33] Chaudhuri A. Matsubara J. A. Cynader M. S. (1995). Neuronal activity in primate visual cortex assessed by immunostaining for the transcription factor Zif268. Vis. Neurosci. 12, 35–50 10.1017/s095252380000729x 7718501

[B34] Chaudhuri A. Zangenehpour S. Matsubara J. A. Cynader M. S. (1996). Differential expression of neurofilament protein in the visual system of the vervet monkey. Brain Res. 709, 17–26 10.1016/0006-8993(95)01217-6 8869552

[B35] Cholfin J. A. Rubenstein J. L. R. (2007). Genetic regulation of prefrontal cortex development and function. Novartis Found. Symp. 288, 165–173; discussion 173–177, 276–281 10.1002/9780470994030.ch12 18494258

[B36] Chou S.-J. Babot Z. Leingärtner A. Studer M. Nakagawa Y. O’Leary D. D. M. (2013). Geniculocortical input drives genetic distinctions between primary and higher-order visual areas. Science 340, 1239–1242 10.1126/science.1232806 23744949 PMC3851411

[B37] Crish S. D. Dengler-Crish C. M. Catania K. C. (2006). Central visual system of the naked mole-rat (Heterocephalus glaber). Anat. Rec. A Discov. Mol. Cell. Evol. Biol. 288, 205–212 10.1002/ar.a.20288 16419086

[B38] Deck M. Lokmane L. Chauvet S. Mailhes C. Keita M. Niquille M. (2013). Pathfinding of corticothalamic axons relies on a rendezvous with thalamic projections. Neuron 77, 472–484 10.1016/j.neuron.2012.11.031 23395374 PMC3756696

[B39] Dehay C. Kennedy H. (2007). Cell-cycle control and cortical development. Nat. Rev. Neurosci. 8, 438–450 10.1038/nrn2176 17514197

[B40] Demeulemeester H. Arckens L. Vandesande F. Orban G. A. Heizmann C. W. Pochet R. (1991). Calcium binding proteins and neuropeptides as molecular markers of GABAergic interneurons in the cat visual cortex. Exp. Brain Res. 84, 538–544 10.1007/bf00230966 1864325

[B41] Demeulemeester H. Vandesande F. Orban G. A. Brandon C. Vanderhaeghen J. J. (1988). Heterogeneity of GABAergic cells in cat visual cortex. J. Neurosci. 8, 988–1000 2894415 10.1523/JNEUROSCI.08-03-00988.1988 PMC6569246

[B42] Depaepe V. Suarez-Gonzalez N. Dufour A. Passante L. Gorski J. A. Jones K. R. (2005). Ephrin signalling controls brain size by regulating apoptosis of neural progenitors. Nature 435, 1244–1250 10.1038/nature03651 15902206

[B43] Dimidschstein J. Passante L. Dufour A. van den Ameele J. Tiberi L. Hrechdakian T. (2013). Ephrin-B1 controls the columnar distribution of cortical pyramidal neurons by restricting their tangential migration. Neuron 79, 1123–1135 10.1016/j.neuron.2013.07.015 24050402

[B44] Donoghue M. J. Rakic P. (1999). Molecular evidence for the early specification of presumptive functional domains in the embryonic primate cerebral cortex. J. Neurosci. 19, 5967–5979 10407035 10.1523/JNEUROSCI.19-14-05967.1999 PMC6783094

[B45] Elsen G. E. Hodge R. D. Bedogni F. Daza R. A. M. Nelson B. R. Shiba N. (2013). The protomap is propagated to cortical plate neurons through an Eomes-dependent intermediate map. Proc. Natl. Acad. Sci. U S A 110, 4081–4086 10.1073/pnas.1209076110 23431145 PMC3593833

[B46] Englund C. Fink A. Lau C. Pham D. Daza R. A. M. Bulfone A. (2005). Pax6, Tbr2 and Tbr1 are expressed sequentially by radial glia, intermediate progenitor cells and postmitotic neurons in developing neocortex. J. Neurosci. 25, 247–251 10.1523/jneurosci.2899-04.2005 15634788 PMC6725189

[B47] Faedo A. Tomassy G. S. Ruan Y. Teichmann H. Krauss S. Pleasure S. J. (2008). COUP-TFI coordinates cortical patterning, neurogenesis and laminar fate and modulates MAPK/ERK, AKT and beta-catenin signaling. Cereb. Cortex 18, 2117–2131 10.1093/cercor/bhm238 18165280 PMC2733307

[B48] Felleman D. J. Van Essen D. C. (1991). Distributed hierarchical processing in the primate cerebral cortex. Cereb. Cortex 1, 1–47 10.1093/cercor/1.1.1 1822724

[B49] Feng G. Laskowski M. B. Feldheim D. A. Wang H. Lewis R. Frisén J. (2000). Roles for ephrins in positionally selective synaptogenesis between motor neurons and muscle fibers. Neuron 25, 295–306 10.1016/s0896-6273(00)80895-8 10719886

[B50] Finlay B. L. Charvet C. J. Bastille I. Cheung D. T. Muniz J. A. P. C. de Lima Silveira L. C. (2014). Scaling the primate lateral geniculate nucleus: niche and neurodevelopment in the regulation of magnocellular and parvocellular cell number and nucleus volume. J. Comp. Neurol. 522, 1839–1857 10.1002/cne.23505 24222647 PMC6556118

[B51] Flanagan J. G. Vanderhaeghen P. (1998). The ephrins and Eph receptors in neural development. Annu. Rev. Neurosci. 21, 309–345 10.1146/annurev.neuro.21.1.309 9530499

[B52] Flenniken A. M. Gale N. W. Yancopoulos G. D. Wilkinson D. G. (1996). Distinct and overlapping expression patterns of ligands for Eph-related receptor tyrosine kinases during mouse embryogenesis. Dev. Biol. 179, 382–401 10.1006/dbio.1996.0269 8903354

[B53] Friedman G. C. O’Leary D. D. (1996). Eph receptor tyrosine kinases and their ligands in neural development. Curr. Opin. Neurobiol. 6, 127–133 10.1016/s0959-4388(96)80018-3 8794058

[B54] Frisén J. Yates P. A. McLaughlin T. Friedman G. C. O’Leary D. D. Barbacid M. (1998). Ephrin-A5 (AL-1/RAGS) is essential for proper retinal axon guidance and topographic mapping in the mammalian visual system. Neuron 20, 235–243 10.1016/s0896-6273(00)80452-3 9491985

[B55] Gale N. W. Yancopoulos G. D. (1997). Ephrins and their receptors: a repulsive topic? Cell Tissue Res. 290, 227–241 10.1007/s004410050927 9321684

[B56] Garel S. Huffman K. J. Rubenstein J. L. R. (2003). Molecular regionalization of the neocortex is disrupted in Fgf8 hypomorphic mutants. Development 130, 1903–1914 10.1242/dev.00416 12642494

[B57] Gelman D. M. Martini F. J. Nóbrega-Pereira S. Pierani A. Kessaris N. Marín O. (2009). The embryonic preoptic area is a novel source of cortical GABAergic interneurons. J. Neurosci. 29, 9380–9389 10.1523/JNEUROSCI.0604-09.2009 19625528 PMC6665570

[B58] Gertz C. C. Lui J. H. LaMonica B. E. Wang X. Kriegstein A. R. (2014). Diverse behaviors of outer radial glia in developing ferret and human cortex. J. Neurosci. 34, 2559–2570 10.1523/JNEUROSCI.2645-13.2014 24523546 PMC3921426

[B59] Giger R. J. Pasterkamp R. J. Heijnen S. Holtmaat A. J. Verhaagen J. (1998). Anatomical distribution of the chemorepellent semaphorin III/collapsin-1 in the adult rat and human brain: predominant expression in structures of the olfactory-hippocampal pathway and the motor system. J. Neurosci. Res. 52, 27–42 10.1002/(sici)1097-4547(19980401)52:1<27::aid-jnr4>3.0.co;2-m 9556027

[B60] Goldman-Rakic P. S. (1988). Topography of cognition: parallel distributed networks in primate association cortex. Annu. Rev. Neurosci. 11, 137–156 10.1146/annurev.neuro.11.1.137 3284439

[B61] Goldshmit Y. Homman-Ludiye J. Bourne J. A. (2014). EphA4 is associated with multiple cell types in the marmoset primary visual cortex throughout the lifespan. Eur. J. Neurosci. 39, 1419–1428 10.1111/ejn.12514 24611983

[B62] Grove E. A. Fukuchi-Shimogori T. (2003). Generating the cerebral cortical area map. Annu. Rev. Neurosci. 26, 355–380 10.1146/annurev.neuro.26.041002.131137 14527269

[B63] Gulisano M. Broccoli V. Pardini C. Boncinelli E. (1996). Emx1 and Emx2 show different patterns of expression during proliferation and differentiation of the developing cerebral cortex in the mouse. Eur. J. Neurosci. 8, 1037–1050 10.1111/j.1460-9568.1996.tb01590.x 8743751

[B64] Gutierrez C. Yaun A. Cusick C. G. (1995). Neurochemical subdivisions of the inferior pulvinar in macaque monkeys. J. Comp. Neurol. 363, 545–562 10.1002/cne.903630404 8847417

[B65] Hall M. I. Kamilar J. M. Kirk E. C. (2012). Eye shape and the nocturnal bottleneck of mammals. Proc. Biol. Sci. 279, 4962–4968 10.1098/rspb.2012.2258 23097513 PMC3497252

[B66] Hansen D. V. Lui J. H. Parker P. R. L. Kriegstein A. R. (2010). Neurogenic radial glia in the outer subventricular zone of human neocortex. Nature 464, 554–561 10.1038/nature08845 20154730

[B67] Hassiotis M. Paxinos G. Ashwell K. W. S. (2004). Cyto- and chemoarchitecture of the cerebral cortex of the Australian echidna (Tachyglossus aculeatus). I. Areal organization. J. Comp. Neurol. 475, 493–517 10.1002/cne.20193 15236232

[B68] Hattori M. Osterfield M. Flanagan J. G. (2000). Regulated cleavage of a contact-mediated axon repellent. Science 289, 1360–1365 10.1126/science.289.5483.1360 10958785

[B69] Haubensak W. Attardo A. Denk W. Huttner W. B. (2004). Neurons arise in the basal neuroepithelium of the early mammalian telencephalon: a major site of neurogenesis. Proc. Natl. Acad. Sci. U S A 101, 3196–3201 10.1073/pnas.0308600100 14963232 PMC365766

[B70] Helmbacher F. Schneider-Maunoury S. Topilko P. Tiret L. Charnay P. (2000). Targeting of the EphA4 tyrosine kinase receptor affects dorsal/ventral pathfinding of limb motor axons. Development 127, 3313–3324 10.1242/dev.1498 10887087

[B71] Hendrickson A. E. Van Brederode J. F. Mulligan K. A. Celio M. R. (1991). Development of the calcium-binding protein parvalbumin and calbindin in monkey striate cortex. J. Comp. Neurol. 307, 626–646 10.1002/cne.903070409 1651352

[B72] Hendry S. H. Jones E. G. Hockfield S. McKay R. D. (1988). Neuronal populations stained with the monoclonal antibody Cat-301 in the mammalian cerebral cortex and thalamus. J. Neurosci. 8, 518–542 3339429 10.1523/JNEUROSCI.08-02-00518.1988 PMC6569291

[B73] Hernández-Miranda L. R. Cariboni A. Faux C. Ruhrberg C. Cho J. H. Cloutier J.-F. (2011). Robo1 regulates Semaphorin signaling to guide the migration of cortical interneurons through the ventral forebrain. J. Neurosci. 31, 6174–6187 10.1523/JNEUROSCI.5464-10.2011 21508241 PMC3088089

[B74] Hockfield S. Tootell R. B. Zaremba S. (1990). Molecular differences among neurons reveal an organization of human visual cortex. Proc. Natl. Acad. Sci. U S A 87, 3027–3031 10.1073/pnas.87.8.3027 2183223 PMC53827

[B75] Hof P. R. Nimchinsky E. A. Morrison J. H. (1995). Neurochemical phenotype of corticocortical connections in the macaque monkey: quantitative analysis of a subset of neurofilament protein-immunoreactive projection neurons in frontal, parietal, temporal and cingulate cortices. J. Comp. Neurol. 362, 109–133 10.1002/cne.903620107 8576425

[B76] Hoffman P. N. Cleveland D. W. Griffin J. W. Landes P. W. Cowan N. J. Price D. L. (1987). Neurofilament gene expression: a major determinant of axonal caliber. Proc. Natl. Acad. Sci. U S A 84, 3472–3476 10.1073/pnas.84.10.3472 3472217 PMC304893

[B77] Holland S. J. Gale N. W. Mbamalu G. Yancopoulos G. D. Henkemeyer M. Pawson T. (1996). Bidirectional signalling through the EPH-family receptor Nuk and its transmembrane ligands. Nature 383, 722–725 10.1038/383722a0 8878483

[B78] Homman-Ludiye J. Bourne J. A. (2013). The guidance molecule Semaphorin3A is differentially involved in the arealization of the mouse and primate neocortex. Cereb. Cortex [Epub ahead of print]. 10.1093/cercor/bht141 23709645

[B79] Homman-Ludiye J. Manger P. R. Bourne J. A. (2010). Immunohistochemical parcellation of the ferret (Mustela putorius) visual cortex reveals substantial homology with the cat (Felis catus). J. Comp. Neurol. 518, 4439–4462 10.1002/cne.22465 20853515

[B80] Huberman A. D. Murray K. D. Warland D. K. Feldheim D. A. Chapman B. (2005). Ephrin-as mediate targeting of eye-specific projections to the lateral geniculate nucleus. Nat. Neurosci. 8, 1013–1021 10.1038/nn1505 16025110 PMC2652399

[B81] Innocenti G. M. Manger P. R. Masiello I. Colin I. Tettoni L. (2002). Architecture and callosal connections of visual areas 17, 18, 19 and 21 in the ferret (Mustela putorius). Cereb. Cortex 12, 411–422 10.1093/cercor/12.4.411 11884356

[B82] Joshi P. S. Molyneaux B. J. Feng L. Xie X. Macklis J. D. Gan L. (2008). Bhlhb5 regulates the postmitotic acquisition of area identities in layers II-V of the developing neocortex. Neuron 60, 258–272 10.1016/j.neuron.2008.08.006 18957218 PMC2643370

[B83] Kaas J. H. (1995). “The segregation of function in the nervous system: why do sensory systems have so many subdivisions?,” in Contributions to Sensory Physiology, ed Neff W. P. (New York, NY: Elsevier Academic Press), 201–240 10.1016/B978-0-12-151807-3.50012-4

[B84] Kaas J. H. (1997). Topographic maps are fundamental to sensory processing. Brain Res. Bull. 44, 107–112 10.1016/s0361-9230(97)00095-6 9292198

[B85] Kaas J. H. Guillery R. W. Allman J. M. (1972). Some principles of organization in the dorsal lateral geniculate nucleus. Brain Behav. Evol. 6, 253–299 10.1159/000123713 4196831

[B86] Kaas J. H. Lyon D. C. (2007). Pulvinar contributions to the dorsal and ventral streams of visual processing in primates. Brain Res. Rev. 55, 285–296 10.1016/j.brainresrev.2007.02.008 17433837 PMC2100380

[B87] Kantor D. B. Chivatakarn O. Peer K. L. Oster S. F. Inatani M. Hansen M. J. (2004). Semaphorin 5A is a bifunctional axon guidance cue regulated by heparan and chondroitin sulfate proteoglycans. Neuron 44, 961–975 10.1016/j.neuron.2004.12.002 15603739

[B88] Kawaguchi A. Ikawa T. Kasukawa T. Ueda H. R. Kurimoto K. Saitou M. (2008). Single-cell gene profiling defines differential progenitor subclasses in mammalian neurogenesis. Development 135, 3113–3124 10.1242/dev.022616 18725516

[B89] Kawasaki H. Iwai L. Tanno K. (2012). Rapid and efficient genetic manipulation of gyrencephalic carnivores using in utero electroporation. Mol. Brain 5:24 10.1186/1756-6606-5-24 22716093 PMC3460770

[B90] Kolodkin A. L. Levengood D. V. Rowe E. G. Tai Y. T. Giger R. J. Ginty D. D. (1997). Neuropilin is a semaphorin III receptor. Cell 90, 753–762 10.1016/S0092-8674(00)80535-8 9288754

[B91] Kolodkin A. L. Matthes D. J. Goodman C. S. (1993). The semaphorin genes encode a family of transmembrane and secreted growth cone guidance molecules. Cell 75, 1389–1399 10.1016/0092-8674(93)90625-z 8269517

[B92] Kowalczyk T. Pontious A. Englund C. Daza R. A. M. Bedogni F. Hodge R. (2009). Intermediate neuronal progenitors (basal progenitors) produce pyramidal-projection neurons for all layers of cerebral cortex. Cereb. Cortex 19, 2439–2450 10.1093/cercor/bhn260 19168665 PMC2742596

[B93] Kravitz D. J. Saleem K. S. Baker C. I. Mishkin M. (2011). A new neural framework for visuospatial processing. Nat. Rev. Neurosci. 12, 217–230 10.1038/nrn3008 21415848 PMC3388718

[B94] Kriegstein A. R. Noctor S. C. (2004). Patterns of neuronal migration in the embryonic cortex. Trends Neurosci. 27, 392–399 10.1016/j.tins.2004.05.001 15219738

[B95] Krishna K. Nuernberger M. Weth F. Redies C. (2009). Layer-Specific expression of multiple cadherins in the developing visual cortex (V1) of the ferret. Cereb. Cortex 19, 388–401 10.1093/cercor/bhn090 18534988

[B96] Krubitzer L. Hunt D. (2007). Captured in the net of space and time: understanding cortical field evolution. Evol. Nervous Syst. Mammals 3, 49–72 10.1016/b0-12-370878-8/00058-6

[B97] Krüger M. Braun T. (2002). The neuronal basic helix-loop-helix transcription factor NSCL-1 is dispensable for normal neuronal development. Mol. Cell. Biol. 22, 792–800 10.1128/mcb.22.3.792-800.2002 11784856 PMC133555

[B98] Kuljis R. O. Rakic P. (1989a). Distribution of neuropeptide Y-containing perikarya and axons in various neocortical areas in the macaque monkey. J. Comp. Neurol. 280, 383–392 10.1002/cne.902800305 2918100

[B99] Kuljis R. O. Rakic P. (1989b). Multiple types of neuropeptide Y-containing neurons in primate neocortex. J. Comp. Neurol. 280, 393–409 10.1002/cne.902800306 2918101

[B100] Kutschera S. Weber H. Weick A. De Smet F. Genove G. Takemoto M. (2011). Differential endothelial transcriptomics identifies semaphorin 3G as a vascular class 3 semaphorin. Arterioscler. Thromb. Vasc. Biol. 31, 151–159 10.1161/ATVBAHA.110.215871 20947821

[B101] Laramée M.-E. Bronchti G. Boire D. (2013b). Primary visual cortex projections to extrastriate cortices in enucleated and anophthalmic mice. Brain Struct. Funct. 1–20 10.1007/s00429-013-0623-6 23942645

[B102] Laramée M. E. Rockland K. S. Prince S. Bronchti G. Boire D. (2013a). Principal component and cluster analysis of layer V pyramidal cells in visual and non-visual cortical areas projecting to the primary visual cortex of the mouse. Cereb. Cortex 23, 714–728 10.1093/cercor/bhs060 22426333

[B103] Lawson S. N. Waddell P. J. (1991). Soma neurofilament immunoreactivity is related to cell size and fibre conduction velocity in rat primary sensory neurons. J. Physiol. 435, 41–63 1770443 10.1113/jphysiol.1991.sp018497 PMC1181449

[B196] Lebrand C. Cases O. Adelbrecht C. Doye A. Alvarez C. Mestikawy E. L. (1996). Transient uptake and storage of serotonin in developing thalamic neurons. Neuron 77, 823–835 10.1016/S0896-6273(00)80215-9 8938116

[B104] Lehigh K. M. Leonard C. E. Baranoski J. Donoghue M. J. (2013). Parcellation of the thalamus into distinct nuclei reflects EphA expression and function. Gene Expr. Patterns 13, 454–463 10.1016/j.gep.2013.08.002 24036135 PMC3839050

[B105] Lein E. S. Hawrylycz M. J. Ao N. Ayres M. Bensinger A. Bernard A. (2007). Genome-wide atlas of gene expression in the adult mouse brain. Nature 445, 168–176 10.1038/nature05453 17151600

[B106] Letinic K. Zoncu R. Rakic P. (2002). Origin of GABAergic neurons in the human neocortex. Nature 417, 645–649 10.1038/nature00779 12050665

[B107] Li H. Fertuzinhos S. Mohns E. Hnasko T. S. Verhage M. Edwards R. (2013). Laminar and columnar development of barrel cortex relies on thalamocortical neurotransmission. Neuron 79, 970–986 10.1016/j.neuron.2013.06.043 24012009 PMC3768017

[B108] Little G. E. López-Bendito G. Rünker A. E. García N. Piñon M. C. Chédotal A. (2009). Specificity and plasticity of thalamocortical connections in Sema6A mutant mice. PLoS Biol. 7:e98 10.1371/journal.pbio.1000098 19402755 PMC2672616

[B109] Liu Q. Dwyer N. D. O’Leary D. D. (2000). Differential expression of COUP-TFI, CHL1 and two novel genes in developing neocortex identified by differential display PCR. J. Neurosci. 20, 7682–7690 11027229 10.1523/JNEUROSCI.20-20-07682.2000 PMC6772850

[B110] López-Bendito G. Molnár Z. (2003). Thalamocortical development: how are we going to get there? Nat. Rev. Neurosci. 4, 276–289 10.1038/nrn1075 12671644

[B111] Lukaszewicz A. Savatier P. Cortay V. Giroud P. Huissoud C. Berland M. (2005). G1 phase regulation, area-specific cell cycle control and cytoarchitectonics in the primate cortex. Neuron 47, 353–364 10.1016/j.neuron.2005.06.032 16055060 PMC1890568

[B112] Lyford G. L. Yamagata K. Kaufmann W. E. Barnes C. A. Sanders L. K. Copeland N. G. (1995). Arc, a growth factor and activity-regulated gene, encodes a novel cytoskeleton-associated protein that is enriched in neuronal dendrites. Neuron 14, 433–445 10.1016/0896-6273(95)90299-6 7857651

[B113] Lyon D. C. Jain N. Kaas J. H. (2003a). The visual pulvinar in tree shrews I. Multiple subdivisions revealed through acetylcholinesterase and Cat-301 chemoarchitecture. J. Comp. Neurol. 467, 593–606 10.1002/cne.10939 14624491

[B114] Lyon D. C. Jain N. Kaas J. H. (2003b). The visual pulvinar in tree shrews II. Projections of four nuclei to areas of visual cortex. J. Comp. Neurol. 467, 607–627 10.1002/cne.10940 14624492

[B115] Ma T. Wang C. Wang L. Zhou X. Tian M. Zhang Q. (2013). Subcortical origins of human and monkey neocortical interneurons. Nat. Neurosci. 16, 1588–1597 10.1038/nn.3536 24097041

[B116] Mallamaci A. Iannone R. Briata P. Pintonello L. Mercurio S. Boncinelli E. (1998). EMX2 protein in the developing mouse brain and olfactory area. Mech. Dev. 77, 165–172 10.1016/s0925-4773(98)00141-5 9831645

[B117] Manger P. R. (2005). Establishing order at the systems level in mammalian brain evolution. Brain Res. Bull. 66, 282–289 10.1016/j.brainresbull.2005.05.002 16144603

[B118] Manger P. R. Engler G. Moll C. K. E. Engel A. K. (2005). The anterior ectosylvian visual area of the ferret: a homologue for an enigmatic visual cortical area of the cat? Eur. J. Neurosci. 22, 706–714 10.1111/j.1460-9568.2005.04246.x 16101752

[B119] Marín O. Rubenstein J. L. R. (2003). Cell migration in the forebrain. Annu. Rev. Neurosci. 26, 441–483 10.1146/annurev.neuro.26.041002.131058 12626695

[B120] Martínez-Cerdeño V. Cunningham C. L. Camacho J. Antczak J. L. Prakash A. N. Cziep M. E. (2012). Comparative analysis of the subventricular zone in rat, ferret and macaque: evidence for an outer subventricular zone in rodents. PLoS One 7:e30178 10.1371/journal.pone.0030178 22272298 PMC3260244

[B121] Matsunaga E. Nambu S. Iriki A. Okanoya K. (2011). Expression pattern of cadherins in the naked mole rat (Heterocephalus glaber) suggests innate cortical diversification of the cerebrum. J. Comp. Neurol. 519, 1736–1747 10.1002/cne.22598 21452207

[B122] Matsunaga E. Nambu S. Oka M. Iriki A. (2013). Differential cadherin expression in the developing postnatal telencephalon of a new world monkey. J. Comp. Neurol. 521, 4027–4060 10.1002/cne.23389 23784870

[B123] Matthews R. T. Kelly G. M. Zerillo C. A. Gray G. Tiemeyer M. Hockfield S. (2002). Aggrecan glycoforms contribute to the molecular heterogeneity of perineuronal nets. J. Neurosci. 22, 7536–7547 12196577 10.1523/JNEUROSCI.22-17-07536.2002 PMC6757962

[B124] McKay R. D. Hockfield S. J. (1982). Monoclonal antibodies distinguish antigenically discrete neuronal types in the vertebrate central nervous system. Proc. Natl. Acad. Sci. U S A 79, 6747–6751 10.1073/pnas.79.21.6747 6959152 PMC347206

[B125] Mishkin M. Ungerleider L. G. (1982). Contribution of striate inputs to the visuospatial functions of parieto-preoccipital cortex in monkeys. Behav. Brain Res. 6, 57–77 10.1016/0166-4328(82)90081-x 7126325

[B126] Molyneaux B. J. Arlotta P. Menezes J. R. L. Macklis J. D. (2007). Neuronal subtype specification in the cerebral cortex. Nat. Rev. Neurosci. 8, 427–437 10.1038/nrn2151 17514196

[B127] Montero V. (1993). Retinotopy of cortical connections between the striate cortex and extrastriate visual areas in the rat. Exp. Brain Res. 94, 1–15 10.1007/bf00230466 8335065

[B128] Mountcastle V. B. (1997). The columnar organization of the neocortex. Brain 120, 701–722 10.1093/brain/120.4.701 9153131

[B129] Nakagawa Y. Johnson J. E. O’Leary D. D. (1999). Graded and areal expression patterns of regulatory genes and cadherins in embryonic neocortex independent of thalamocortical input. J. Neurosci. 19, 10877–10885 10594069 10.1523/JNEUROSCI.19-24-10877.1999 PMC6784968

[B130] Nery S. Fishell G. Corbin J. G. (2002). The caudal ganglionic eminence is a source of distinct cortical and subcortical cell populations. Nat. Neurosci. 5, 1279–1287 10.1038/nn971 12411960

[B131] Nonaka-Kinoshita M. Reillo I. Artegiani B. Martínez-Martínez M. Á. Nelson M. Borrell V. (2013). Regulation of cerebral cortex size and folding by expansion of basal progenitors. EMBO J. 32, 1817–1828 10.1038/emboj.2013.96 23624932 PMC3926188

[B132] Nys J. Aerts J. Ytebrouck E. Vreysen S. Laeremans A. Arckens L. (2014). The cross-modal aspect of mouse visual cortex plasticity induced by monocular enucleation is age dependent. J. Comp. Neurol. 522, 950–970 10.1002/cne.23455 24037705

[B133] Obst K. Bronzel M. Wahle P. (1998). Visual activity is required to maintain the phenotype of supragranular NPY neurons in rat area 17. Eur. J. Neurosci. 10, 1422–1428 10.1046/j.1460-9568.1998.00146.x 9749796

[B134] Obst K. Wahle P. (1995). Areal differences of NPY mRNA-expressing neurons are established in the late postnatal rat visual cortex in vivo, but not in organotypic cultures. Eur. J. Neurosci. 7, 2139–2158 10.1111/j.1460-9568.1995.tb00636.x 8542071

[B135] Oeschger F. M. Wang W.-Z. Lee S. García-Moreno F. Goffinet A. M. Arbonés M. L. (2012). Gene expression analysis of the embryonic subplate. Cereb. Cortex 22, 1343–1359 10.1093/cercor/bhr197 21862448 PMC4972418

[B136] O’Leary D. D. (1989). Do cortical areas emerge from a protocortex? Trends Neurosci. 12, 400–406 10.1016/0166-2236(89)90080-5 2479138

[B137] O’Leary D. D. M. Chou S.-J. Sahara S. (2007). Area patterning of the mammalian cortex. Neuron 56, 252–269 10.1016/j.neuron.2007.10.010 17964244

[B138] O’Leary D. D. Sahara S. (2008). Genetic regulation of arealization of the neocortex. Curr. Opin. Neurobiol. 18, 90–100 10.1016/j.conb.2008.05.011 18524571 PMC2677555

[B139] Paolini M. Sereno M. I. (1998). Direction selectivity in the middle lateral and lateral (ML and L) visual areas in the California ground squirrel. Cereb. Cortex 8, 362–371 10.1093/cercor/8.4.362 9651131

[B140] Polleux F. Morrow T. Ghosh A. (2000). Semaphorin 3A is a chemoattractant for cortical apical dendrites. Nature 404, 567–573 10.1038/35007001 10766232

[B141] Poluch S. Jablonska B. Juliano S. L. (2008). Alteration of interneuron migration in a ferret model of cortical dysplasia. Cereb. Cortex 18, 78–92 10.1093/cercor/bhm032 17443019

[B142] Pontious A. Kowalczyk T. Englund C. Hevner R. F. (2008). Role of intermediate progenitor cells in cerebral cortex development. Dev. Neurosci. 30, 24–32 10.1159/000109848 18075251

[B143] Quinn J. C. Molinek M. Martynoga B. S. Zaki P. A. Faedo A. Bulfone A. (2007). Pax6 controls cerebral cortical cell number by regulating exit from the cell cycle and specifies cortical cell identity by a cell autonomous mechanism. Dev. Biol. 302, 50–65 10.1016/j.ydbio.2006.08.035 16979618 PMC2384163

[B144] Raghanti M. A. Conley T. Sudduth J. Erwin J. M. Stimpson C. D. Hof P. R. (2013). Neuropeptide Y-immunoreactive neurons in the cerebral cortex of humans and other haplorrhine primates. Am. J. Primatol. 75, 415–424 10.1002/ajp.22082 23042407 PMC3560302

[B145] Rakic P. (1988). Specification of cerebral cortical areas. Science 241, 170–176 10.1126/science.3291116 3291116

[B146] Rakic P. (1995). A small step for the cell, a giant leap for mankind: a hypothesis of neocortical expansion during evolution. Trends Neurosci. 18, 383–388 10.1016/0166-2236(95)93934-p 7482803

[B147] Rakic P. (2002). Neurogenesis in adult primates. Prog. Brain Res. 138, 3–14 10.1016/s0079-6123(02)38067-1 12432759

[B148] Rakic P. Ayoub A. E. Breunig J. J. Dominguez M. H. (2009). Decision by division: making cortical maps. Trends Neurosci. 32, 291–301 10.1016/j.tins.2009.01.007 19380167 PMC3601545

[B149] Redies C. Takeichi M. (1996). Cadherins in the developing central nervous system: an adhesive code for segmental and functional subdivisions. Dev. Biol. 180, 413–423 10.1006/dbio.1996.0315 8954714

[B150] Reillo I. de Juan Romero C. García-Cabezas M. Á. Borrell V. (2011). A role for intermediate radial glia in the tangential expansion of the mammalian cerebral cortex. Cereb. Cortex 21, 1674–1694 10.1093/cercor/bhq238 21127018

[B151] Rockel A. J. Hiorns R. W. Powell T. P. (1980). The basic uniformity in structure of the neocortex. Brain 103, 221–244 10.1093/brain/103.2.221 6772266

[B152] Ross C. F. (2000). The origin of Anthropoidea. Annu. Rev. Anthropol. 29, 147–194 10.1146/annurev.anthro.29.1.147

[B153] Rudy B. Fishell G. Lee S. Hjerling-Leffler J. (2011). Three groups of interneurons account for nearly 100% of neocortical GABAergic neurons. Dev. Neurobiol. 71, 45–61 10.1002/dneu.20853 21154909 PMC3556905

[B154] Saito T. Nakatsuji N. (2001). Efficient gene transfer into the embryonic mouse brain using in vivo electroporation. Dev. Biol. 240, 237–246 10.1006/dbio.2001.0439 11784059

[B155] Sansom S. N. Griffiths D. S. Faedo A. Kleinjan D.-J. Ruan Y. Smith J. (2009). The level of the transcription factor Pax6 is essential for controlling the balance between neural stem cell self-renewal and neurogenesis. PLoS Genet. 5:e1000511 10.1371/journal.pgen.1000511 19521500 PMC2686252

[B156] Sessa A. Mao C.-A. Hadjantonakis A.-K. Klein W. H. Broccoli V. (2008). Tbr2 directs conversion of radial glia into basal precursors and guides neuronal amplification by indirect neurogenesis in the developing neocortex. Neuron 60, 56–69 10.1016/j.neuron.2008.09.028 18940588 PMC2887762

[B157] Sherwood C. C. Stimpson C. D. Butti C. Bonar C. J. Newton A. L. Allman J. M. (2009). Neocortical neuron types in Xenarthra and Afrotheria: implications for brain evolution in mammals. Brain Struct. Funct. 213, 301–328 10.1007/s00429-008-0198-9 19011898

[B158] Shitamukai A. Konno D. Matsuzaki F. (2011). Oblique radial glial divisions in the developing mouse neocortex induce self-renewing progenitors outside the Germinal zone that resemble primate outer subventricular zone progenitors. J. Neurosci. 31, 3683–3695 10.1523/jneurosci.4773-10.2011 21389223 PMC6622781

[B159] Sia Y. Bourne J. A. (2008). The rat temporal association cortical area 2 (Te2) comprises two subdivisions that are visually responsive and develop independently. Neuroscience 156, 118–128 10.1016/j.neuroscience.2008.07.002 18674594

[B160] Smart I. H. M. Dehay C. Giroud P. Berland M. Kennedy H. (2002). Unique morphological features of the proliferative zones and postmitotic compartments of the neural epithelium giving rise to striate and extrastriate cortex in the monkey. Cereb. Cortex 12, 37–53 10.1093/cercor/12.1.37 11734531 PMC1931430

[B161] Stahl R. Walcher T. de Juan Romero C. Pilz G. A. Cappello S. Irmler M. (2013). Trnp1 regulates expansion and folding of the Mammalian cerebral cortex by control of radial glial fate. Cell 153, 535–549 10.1016/j.cell.2013.03.027 23622239

[B162] Sternberger L. A. Sternberger N. H. (1983). Monoclonal antibodies distinguish phosphorylated and nonphosphorylated forms of neurofilaments in situ. Proc. Natl. Acad. Sci. U S A 80, 6126–6130 10.1073/pnas.80.19.6126 6577472 PMC534374

[B163] Steinberg M. S. Takeichi M. (1994). Experimental specification of cell sorting, tissue spreading and specific spatial patterning by quantitative differences in cadherin expression. Proc. Natl. Acad. Sci. U S A 91, 206–209 10.1073/pnas.91.1.206 8278366 PMC42915

[B164] Stoykova A. Gruss P. (1994). Roles of Pax-genes in developing and adult brain as suggested by expression patterns. J. Neurosci. 14, 1395–1412 8126546 10.1523/JNEUROSCI.14-03-01395.1994 PMC6577564

[B165] Sultan K. T. Brown K. N. Shi S.-H. (2013). Production and organization of neocortical interneurons. Front. Cell. Neurosci. 7:221 10.3389/fncel.2013.00221 24312011 PMC3836051

[B166] Takeichi M. (2007). The cadherin superfamily in neuronal connections and interactions. Nat. Rev. Neurosci. 8, 11–20 10.1038/nrn2043 17133224

[B167] Teo L. Homman-Ludiye J. Rodger J. Bourne J. A. (2012). Discrete ephrin-B1 expression by specific layers of the primate retinogeniculostriate system continues throughout postnatal and adult life. J. Comp. Neurol. 520, 2941–2956 10.1002/cne.23077 22778007

[B168] Tian L. Hires S. A. Mao T. Huber D. Chiappe M. E. Chalasani S. H. (2009). Imaging neural activity in worms, flies and mice with improved GCaMP calcium indicators. Nat. Methods 6, 875–881 10.1038/nmeth.1398 19898485 PMC2858873

[B169] Tohmi M. Meguro R. Tsukano H. Hishida R. Shibuki K. (2014). The extrageniculate visual pathway generates distinct response properties in the higher visual areas of mice. Curr. Biol. 24, 587–597 10.1016/j.cub.2014.01.061 24583013

[B170] Tomioka R. (2006). Improved Golgi-like visualization in retrogradely projecting neurons after EGFP-adenovirus infection in adult rat and monkey. J. Histochem. Cytochem. 54, 539–548 10.1369/jhc.5a6838.2005 16344324

[B171] Tomita K. Sperling M. Cambridge S. B. Bonhoeffer T. Hübener M. (2013). A molecular correlate of ocular dominance columns in the developing mammalian visual cortex. Cereb. Cortex 23, 2531–2541 10.1093/cercor/bhs232 22892426

[B172] Ungerleider L. G. Haxby J. V. (1994). ‘What’ and “where” in the human brain. Curr. Opin. Neurobiol. 4, 157–165 10.1016/0959-4388(94)90066-3 8038571

[B173] Ungerleider L. G. Mishkin M. (1982). “Two cortical visual systems,” in Analysis of Visual Behavior, eds Ingle D. J. Goodale M. A. Mansfield R. J. W. (Cambridge, MA: MIT Press), 549–586

[B174] Van Brussel L. Gerits A. Arckens L. (2011). Evidence for cross-modal plasticity in adult mouse visual cortex following monocular enucleation. Cereb. Cortex 21, 2133–2146 10.1093/cercor/bhq286 21310780

[B175] Van der Gucht E. Hof P. R. Van Brussel L. Burnat K. Arckens L. (2007). Neurofilament protein and neuronal activity markers define regional architectonic parcellation in the mouse visual cortex. Cereb. Cortex 17, 2805–2819 10.1093/cercor/bhm012 17337746

[B176] van der Gucht E. Vandesande F. Arckens L. (2001). Neurofilament protein: a selective marker for the architectonic parcellation of the visual cortex in adult cat brain. J. Comp. Neurol. 441, 345–368 10.1002/cne.1416 11745654

[B177] Vue T. Y. Lee M. Tan Y. E. Werkhoven Z. Wang L. Nakagawa Y. (2013). Thalamic control of neocortical area formation in mice. J. Neurosci. 33, 8442–8453 10.1523/JNEUROSCI.5786-12.2013 23658181 PMC3732791

[B178] Wagor E. Mangini N. J. Pearlman A. L. (1980). Retinotopic organization of striate and extrastriate visual cortex in the mouse. J. Comp. Neurol. 193, 187–202 10.1002/cne.901930113 6776164

[B179] Walther C. Gruss P. (1991). Pax-6, a murine paired box gene, is expressed in the developing CNS. Development 113, 1435–1449 1687460 10.1242/dev.113.4.1435

[B180] Wang Q. Burkhalter A. (2007). Area map of mouse visual cortex. J. Comp. Neurol. 502, 339–357 10.1002/cne.21286 17366604

[B181] Wang Q. Sporns O. Burkhalter A. (2012). Network analysis of corticocortical connections reveals ventral and dorsal processing streams in mouse visual cortex. J. Neurosci. 32, 4386–4399 10.1523/jneurosci.6063-11.2012 22457489 PMC3328193

[B182] Wang X. Tsai J.-W. Lamonica B. Kriegstein A. R. (2011). A new subtype of progenitor cell in the mouse embryonic neocortex. Nat. Neurosci. 14, 555–561 10.1038/nn.2807 21478886 PMC3083489

[B183] Watakabe A. Hirokawa J. Ichinohe N. Ohsawa S. Kaneko T. Rockland K. S. (2012). Area-specific substratification of deep layer neurons in the rat cortex. J. Comp. Neurol. 520, 3553–3573 10.1002/cne.23160 22678985

[B184] Wong P. Kaas J. H. (2008). Architectonic subdivisions of neocortex in the gray squirrel (Sciurus carolinensis). Anat. Rec. (Hoboken) 291, 1301–1333 10.1002/ar.20758 18780299 PMC2908424

[B185] Wong P. Kaas J. H. (2009). An architectonic study of the neocortex of the short-tailed opossum (Monodelphis domestica). Brain Behav. Evol. 73, 206–228 10.1159/000225381 19546531 PMC3710711

[B186] Wong-Riley M. (1979). Changes in the visual system of monocularly sutured or enucleated cats demonstrable with cytochrome oxidase histochemistry. Brain Res. 171, 11–28 Available at: http://eutils.ncbi.nlm.nih.gov/entrez/eutils/elink.fcgi?dbfrom=pubmed&id=223730&retmode=ref&cmd=prlinks 10.1016/0006-8993(79)90728-5 223730

[B187] Yun M. E. Johnson R. R. Antic A. Donoghue M. J. (2003). EphA family gene expression in the developing mouse neocortex: regional patterns reveal intrinsic programs and extrinsic influence. J. Comp. Neurol. 456, 203–216 10.1002/cne.10498 12528186

[B188] Zangenehpour S. Chaudhuri A. (2002). Differential induction and decay curves of c-fos and zif268 revealed through dual activity maps. Brain Res. Mol. Brain Res. 109, 221–225 10.1016/s0169-328x(02)00556-9 12531532

[B189] Zaremba S. Guimaraes A. Kalb R. G. Hockfield S. (1989). Characterization of an activity-dependent, neuronal surface proteoglycan identified with monoclonal antibody Cat-301. Neuron 2, 1207–1219 10.1016/0896-6273(89)90305-x 2624746

[B190] Zecevic N. Chen Y. Filipovic R. (2005). Contributions of cortical subventricular zone to the development of the human cerebral cortex. J. Comp. Neurol. 491, 109–122 10.1002/cne.20714 16127688 PMC2628573

[B191] Zembrzycki A. Griesel G. Stoykova A. Mansouri A. (2007). Genetic interplay between the transcription factors Sp8 and Emx2 in the patterning of the forebrain. Neural Dev. 2:8 10.1186/1749-8104-2-8 17470284 PMC1868949

[B192] Zeng H. Shen E. H. Hohmann J. G. Oh S. W. Bernard A. Royall J. J. (2012). Large-scale cellular-resolution gene profiling in human neocortex reveals species-specific molecular signatures. Cell 149, 483–496 10.1016/j.cell.2012.02.052 22500809 PMC3328777

[B193] Zilles K. Palomero-Gallagher N. Amunts K. (2013). Development of cortical folding during evolution and ontogeny. Trends Neurosci. 36, 275–284 10.1016/j.tins.2013.01.006 23415112

[B194] Zimmer G. Rudolph J. Landmann J. Gerstmann K. Steinecke A. Gampe C. (2011). Bidirectional ephrinB3/EphA4 signaling mediates the segregation of medial ganglionic eminence- and preoptic area-derived interneurons in the deep and superficial migratory stream. J. Neurosci. 31, 18364–18380 10.1523/jneurosci.4690-11.2011 22171039 PMC6623906

[B195] Zimmer G. Schanuel S. M. Bürger S. Weth F. Steinecke A. Bolz J. (2010). Chondroitin sulfate acts in concert with semaphorin 3A to guide tangential migration of cortical interneurons in the ventral telencephalon. Cereb. Cortex 20, 2411–2422 10.1093/cercor/bhp309 20071458

